# Experimental and AI-based prediction of a solar air heater with novel recycled interlocking channel fins

**DOI:** 10.1038/s41598-026-61438-1

**Published:** 2026-07-14

**Authors:** M. Koraiem M. Handawy, Hisham Maher, Hamada Mohamed Abdelmotalib

**Affiliations:** Mechanical Power Engineering and Energy Department, Faculty of Engineering, Minia University, Minia, 61519 Egypt

**Keywords:** Artificial intelligence, Solar air heater, Recycled aluminum, Fins, Energy, Energy, Energy science and technology, Engineering

## Abstract

Solar air heaters are a promising method used for drying and heating applications owing to their low operating costs and simple design. However, these solar air heaters exhibit limited thermal performance due to the low heat transfer coefficient between the absorber and the air passing through the duct, resulting in a decrease in thermal and exergy efficiency. The present work aims to address this issue by conducting a single-pass solar air heater with novel recycled aluminum interlocking channel fins, along with using artificial intelligence approaches to predict thermal and exergy efficiencies. The novelty of the present study is represented by integrating a comprehensive experimental assessment using the 4E analysis (Energy, Exergy, Economic, and Environmental analysis) with the development and comparison of artificial neural network (ANN) and deep neural network (DNN) models to predict efficiency under different operating conditions. Two solar air heaters were tested: a conventional (C-SAH) and a modified finned (M-SAH) heater, under natural and forced convection conditions at mass flow rates of 0.0046, 0.008, and 0.012 kg/s. The study findings reveal a significant enhancement in the modified heater. Under natural convection conditions, the outlet air temperature increased from 78 °C to 84 °C for the modified heater. Regarding thermal efficiency, the modified heater exhibited the highest efficiency of 48.6% at a flow rate of 0.012 kg/s. Daily thermal efficiency also increased from 32.91% to 44.36% at the same flow rate. The exergy efficiency reached a maximum of 2.80% for the modified heater. The AI models achieved high predictive performance; the DNN model achieved an R² of 0.924 for thermal efficiency, while the ANN model performed best in predicting exergy efficiency with an R² of 0.971. These results demonstrate the reliability of the proposed AI models in predicting the performance of SAHs. Additionally, the use of recycled aluminum fins significantly enhances the performance of SAHs, offering a low-cost and sustainable tool for solar air heating applications. The integration of artificial intelligence methods enhances the design and operation of highly efficient solar air heater systems.

## Introduction

A major sustainable energy source, solar energy has attracted a lot of study to improve plant thermal efficiency while lowering carbon dioxide emissions and the usage of fossil fuels^1,2^. Additionally, solar systems offer a great deal of potential for generating electricity and heating by using gas or liquid as the working fluid to absorb solar radiation and then transform it into heat. The Solar Air Heater (SAH) model includes a basic conventional system used in a variety of thermal applications at moderate temperatures, including space heating, drying operations, power production, and its contribution to meeting the world’s energy needs^3–6^. However, SAH systems also have some limitations in case of using air as a working fluid, such as low air thermal conductivity, pressure drop inside the collector, a small absorber surface area, and energy losses^7–9^. Employment of these systems and solutions is no longer sufficient, though; control and regulation systems are becoming crucial for developing and implementing strategies that maximize all the variables affecting how new systems and solutions work and operate, as well as in the environments that require these interventions. The optimization of the obtained green energy resources, as well as saving electricity consumption and auxiliary energy requirements, must be the focus of these control and regulation systems^10^.

In solar air heaters (SAHs), the absorber plate receives solar radiation transmitted through the transparent glass cover, converting it into thermal energy. This process increases the temperature of the absorber plate, which subsequently heats the air flowing over it. Heat transfer occurs primarily through convection, raising the air temperature as it absorbs energy from the heated plate. Additionally, thermal radiation emitted by the absorber plate further enhances heat transfer to the surrounding air, improving the overall efficiency of the system. A solar air heater is widely used for its ability to absorb both direct and diffuse solar radiation effectively. These systems are known for their low manufacturing costs, simple structure, durability, reliability, and ease of integration into buildings, making them a cost-effective solution for air heating applications^11^. However, solar air heaters have limitations, such as a low heat transfer coefficient between the absorption plate and the air due to losses of absorbed thermal energy. Also, the decrease in the thermal capacity of the air reduces the thermal efficiency of the solar air heater. As a result, any design of a solar air heater must address these defects in order to avoid undesirable performance. There have been ongoing efforts to improve the SAH design, including enhancing the heat transfer coefficient between the absorber plate and the flowing air^12^. There have been ongoing efforts to improve the SAH design, including enhancing the heat transfer coefficient between the absorber plate and the flowing air^13,14^. Researchers have investigated a variety of methods to improve the thermal performance of SAHs, such as employing multi-pass configurations to optimize airflow^15^ and adding extended surfaces like wire mesh^16^, baffles^17,18^, vortex generators^19^, artificial roughness^20^, and fins^21,22^to improve convective heat transfer between the absorber and the airflow^10^. Because fins can create turbulence and increase surface area, they improve efficiency.

A theoretical analysis of the performance increase by adding fins to the absorber plate was presented by Kumar and Chand^23^. Corrugated fins were integrated into the absorber’s lower side along the fluid flow channel. They concluded that using a 25 mm fin increased thermal performance by a range of 36.2% to 56.6%. The absorber plate’s surface area was expanded by adding fins, which improved the solar air heater’s total thermal efficiency. However, there was also an increase in the air flow’s pressure drop. Solar air heater efficiency can also be increased by having an absorber material with high thermal conductivity and a high air mass flow rate. The effect of operating factors, such as fin length, fin distance, fluid mass flow rate, and types of insolation materials, on the thermo-hydraulic performance and efficiency of the finned absorber of solar air heaters was studied parametrically by Rai et al^24^.. They demonstrated that as the fluid mass flow rate increases, thermal efficiency also increases. An experimental evaluation of the solar air collector’s combined performance with S-shaped ribs with gaps was conducted by Wang et al^25^.. In addition to the solar radiation and air mass flow rates, the design parameters were evaluated, including the size of the rib geometry, such as rib spacing, breadth, and channel height. They demonstrated how the heat transmission between the absorber and the air may be adequately increased by an S-shaped absorber with a gap. Compared to a conventional flat collector, the suggested solar air heater’s thermal performance is 65% better thanks to its “S”-shaped fins. An experimental and theoretical study on the incorporation of paraffin-filled pin-fins as a thermal storage material in the absorber was carried out by Arul Kumar et al^26^. Studies were also conducted on the impact of various meteorological conditions in Coimbatore, India. They concluded that, in comparison to a conventional flat heater, the usage of pin fins with paraffin wax raised the outlet air temperatures by 5 °C for up to three hours. Furthermore, compared to the conventional flat heater, thermo-hydraulic efficiency and exergy efficiency increased by 35% and 15%, respectively. A new design for hybrid solar heating of water and air was presented by Kumar et al^27^.. Both the absorber surface in the water heater and the inner walls of the air ducts in the air heater were roughened using a pressured shot-blasting technique in order to enhance the thermal performance of the suggested hybrid collector. They discovered that at a mass flow rate of 0.01 kg/s, the optimal thermal performance was 51.03%. In order to minimize installation expenses related to water and space heating, in the study conducted by Vengadesan et al^28^., They integrated flat solar collectors to heat water and air. It was found that the energy and maximum energy efficiencies of the integrated heat storage collector were 88.8% and 3.5% higher, respectively, than those of the collector without heat storage at 0.025 kg/s for water flow and 0.0132 kg/s for air mass flow. At a higher flow rate than without heat storage, the overall heat loss coefficient of the combined heat storage collector is 4.2% lower. Table 1 summarizes some modifications used to improve the performance of different types of SAH.

**Table 1:** Summary of various enhancements used to improve the performance of SAH.

Refs.	Studied modification	Main finding
Arul Kumar et al^26^.	Paraffin-filled pin fins integrated with absorber	Thermal storage improved outlet air temperature and maintained heat supply for extended hours after sunset.
Elbrashy et al^29^.	Evacuated tubes filled with storage material	Incorporating storage material enhanced heat retention and increased outlet air temperature compared to non-storage design.
Vengadesan et al^28^.	Integrated collectors with heat storage tubes	Heat storage tubes reduced heat losses and improved overall energy and exergy efficiencies.
Yousef et al.^30^	Tubular SAH with porous material, single and double pass	Porous material and tubular configuration enhanced thermal performance, economic viability, and reduced energy cost compared to flat plate heaters.
Assadeg et al^31^.	Double-pass SAH with fins and PCM	Combined use of fins and PCM significantly enhanced energy efficiency and overall thermal performance over other configurations
Kumar and Chand^23^	Corrugated fins attached to absorber plate	Adding fins significantly increased heat transfer area and improved thermal efficiency.
Rai et al^24^.	Variation of fin length, spacing, and mass flow rate	Thermal efficiency improved with optimized fin geometry and higher air mass flow rates.
Wang et al^25^.	S-shaped ribs with gap on absorber plate	Surface ribs enhanced turbulence and absorber–air heat transfer, leading to higher thermal performance.
Kumar and Rosen^32^	Fins (PV/T double-pass)	Vertical fins in lower channel of double-pass PV/T SAH Fins increased heat transfer area, improved thermal and electrical efficiencies, and significantly reduced cell temperature
Kabeel et al^33^.	Fins/Corrugated absorber	V-corrugated plate achieved the highest outlet air temperature and thermal efficiency due to enhanced convective heat transfer compared with flat and finned plates.
Pachori et al.^34^	Arc-shape roughness collector	The SAH with arc-shaped collector achieved higher efficiency than that of the smooth duct
Pachori et al^35^.	V-shaped artificial roughness	The V-ribbed absorber had higher thermal efficiency than that of the smooth duct by 40%.

In order to optimize a wavy-corrugated solar air collector with PCM storage, Zayed et al^36^.combined experimental testing with SVM-Monte Carlo modeling. Their system achieved 16–24% higher daily efficiency with 4 cm paraffin wax, while the machine learning model predicted performance with R^2^> 0.95 accuracy. This shows the dual potential of wavy absorbers and AI modeling, though their PCM integration differs from the current study’s focus on pure geometric optimization. Ganesh Kumar et al^37^. used ML techniques to analyze the thermal performance of a V-corrugation, shot-blasted absorber plate with black pebble-based heat storage. They employed regression and classification methods to achieve very accurate projections for the Nusselt number and overall performance with a mean error of less than 2%. Artificial neural networks (ANNs) were used by Bhattacharyya et al^38^. to forecast heat transfer and performance in helical corrugated SAH tubes with perforated discs. They were able to predict the Nusselt number and thermo-hydraulic efficiency with a 97% prediction accuracy. Similar to this, Das et al^39^. demonstrated good statistical reliability (R² > 0.99) and prediction errors under ± 5% when using ANN models to estimate the thermal, exergy, and thermo-hydraulic efficiency of jet impingement SAHs across a range of climatic and operational parameters. Using 504 CFD-generated data sets, Singh et al.^40^ conducted another ANN-based study that predicted the thermo-hydraulic performance of SAHs with vertical cylindrical ribs. The study’s maximum performance was 1.43, and it used SHAP analysis to identify the Reynolds number and rib pitch as important determinants.

In order to estimate the performance of ribbed triangular duct SAHs, Nidhul et al^41^. built a machine learning model with artificial neural networks. They achieved prediction errors of less than 3% by training the model on 454 data points from 72 distinct rib configurations, allowing for precise predictions of collector performance and entropy generation without the need for additional testing. Similarly, to forecast the thermal performance of SAHs with C-shaped finned absorber plates, Saravanan et al^42^.used three machine learning models: random forest, linear regression, and K-nearest neighbors. With an accuracy of 98% and an R2 of 0.9783, the RF model fared better than the others, making it the perfect prediction tool for determining thermal efficiency, friction factor, and Nusselt number^43^.

Chebaane et al^44^. used ANFIS neural network model to predict the performance of SAH integrated with PCM and porous foam gradient. The model could accurately predict the performance achieving R^2^of 0.9973. The CFD results of SAH were used to develop and train A MLP neural network model in this study by Hamida et al^45^.. The model was integrated with genetic algorithm (GA) and particle swarm optimization (PSO) algorithms. The study findings reveal that the PSO-based model accurately predicted thermal performance with R^2^ greater than 0.99, while GA exhibited higher accuracy for predicting friction factor with R^2^around 0.999. Zayed et al^46^. employed an SVM-Monte Carlo approach to model the performance of a wavy-corrugated SAH with PCM. The proposed model accurately predicted the performance of the modified SAH with R² greater than 0.95. The effect of different modifications on the operation of the SAH was modeled using an MLP-ANN model. The model was developed using 64 datasets from experimental runs, achieving a lower average error of 1.58%. A DNN model was developed to predict the performance of double-pass SAH integrated with PCM-filled helical finned tubes by Rehman et al.^47^. The model was trained using experimental data from different designs of SAH, achieving higher prediction accuracy with R² of 0.995 and RMSE of 0.018. Despite the large number of studies on solar air heaters, the majority of those studies concern either geometric improvements or traditional performance indicators such as thermal efficiency, while Comprehensive analyses combining advanced designs with both energy and exergy perspectives remain limited. In particular, the coupled effects of operating conditions, absorber–fin arrangements, and multi-parameter interactions on outlet air temperature, thermal performance, and exergy behavior have not been sufficiently quantified using robust AI techniques. Furthermore, previous studies commonly depended on single modeling methods, which limit the generalization ability and predictive reliability of the obtained results. Therefore, an obvious research gap exists in developing a detailed hybrid modeling framework capable of accurately predicting the performance of modified SAH under varying operating conditions. In consideration of this, this work offers a novel comprehensive framework that combines physical experimental work with AI-predictive modeling and multi-objective optimization. The novelty of this work includes: (1) the design and development of novel 3D recycled aluminum interlocking channel fins that improve heat transfer while enhancing sustainability; (2) the development of AI models not merely to predict performance, but also to capture uncover non-linear thermal and exergy dynamics of the system; and (3) the use of the AI models to carried out multi-objective Pareto optimization. The optimization process can determine critical operational variables that maximize net efficiency by balancing thermal gains against parasitic fan losses.

## Experimental method

### Experimental setup

**Fig. 1:**
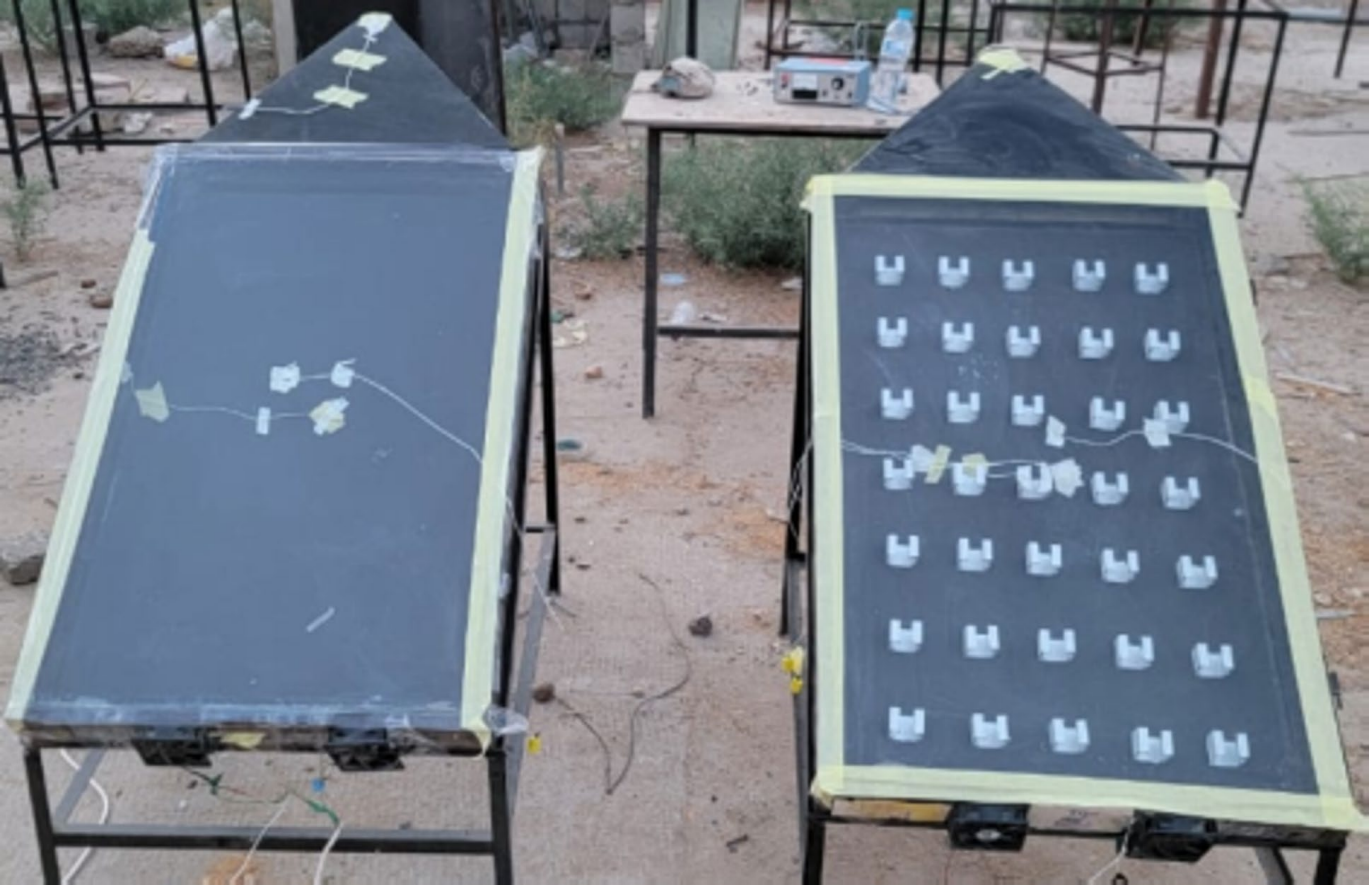
A real photo of experimental setup used in this study.

**Fig. 2:**
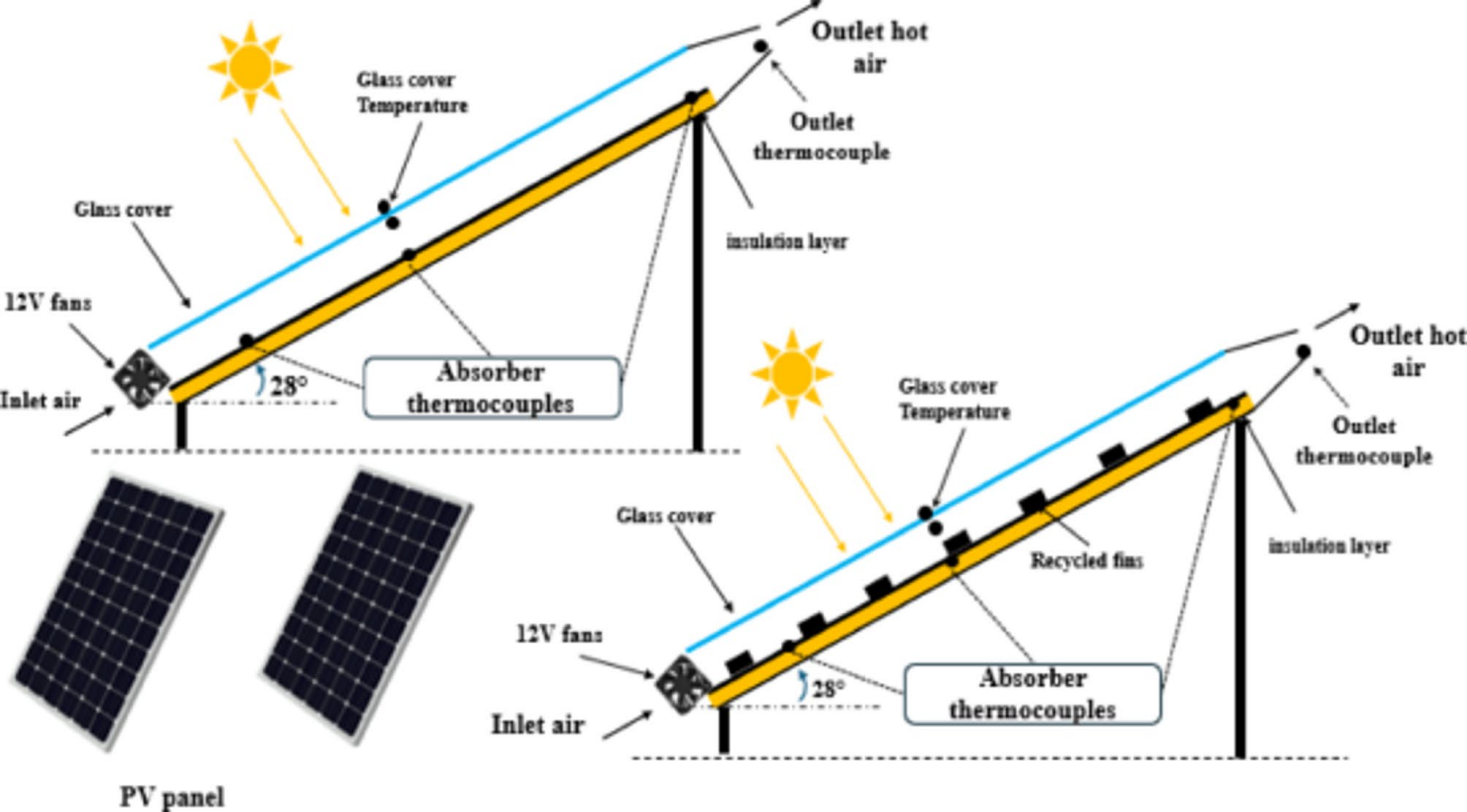
Schematic diagram of the SAH with detailed 2D cross-sectional views of the recycled ICF.

This section describes the experimental setup, as shown in Fig. 1, used to evaluate the improved thermal performance of a modified solar air heater (M-SAH) with recycled aluminum fins, compared to a conventional solar air heater (C-SAH). Two single-pass solar air heaters were designed, fabricated, and installed at the Faculty of Engineering, Minia University, Egypt with a latitude of 28.08° and longitude of 30.73. The experiments were conducted from May 23 to 26, 2025, a period that represents the onset of the peak summer season in Minia, Egypt (latitude 28.08° N). This timeframe was specifically selected to estimate the performance of SAH at maximum solar irradiance, which peaks at around of 1090 W/m², and high ambient temperatures, reaching 41 °C. These conditions represent the most critical peak season for high-load thermal applications, such as industrial space heating and drying of agricultural products, which provides a rigorous reference to identify the maximum thermal improvement of the proposed modifications. The specifications of each heater are shown in Table 2. Each heater consists of a rectangular wooden casing with a 10 cm high air duct, mounted at a 28° incline towards the south. The absorber plate is made of galvanized steel (100 × 60 cm) with a thickness of 2 mm, and beneath it is a 5 cm-thick layer of glass wool insulation. Each heater is covered with 4 mm thick glass, as shown in Fig. 2.

**Table 2:** Specifications of conventional and modified SAH.

Component	Material	Specifications
SAHs, single pass	Wooden	Length 101cm, width 61cm
The air gap	-	10 cm
Cover	Glass, thickness 4mm	100cm*60cm
Absorber plate	Galvanized steel	100 cm*60 cm
Fins	Aluminum	Polygonal U-shape, with 4cm*4cm*4cm
Inclined SAH angle		28°
Insulation	Glass wool	Thickness 5 cm

**Fig. 3:**
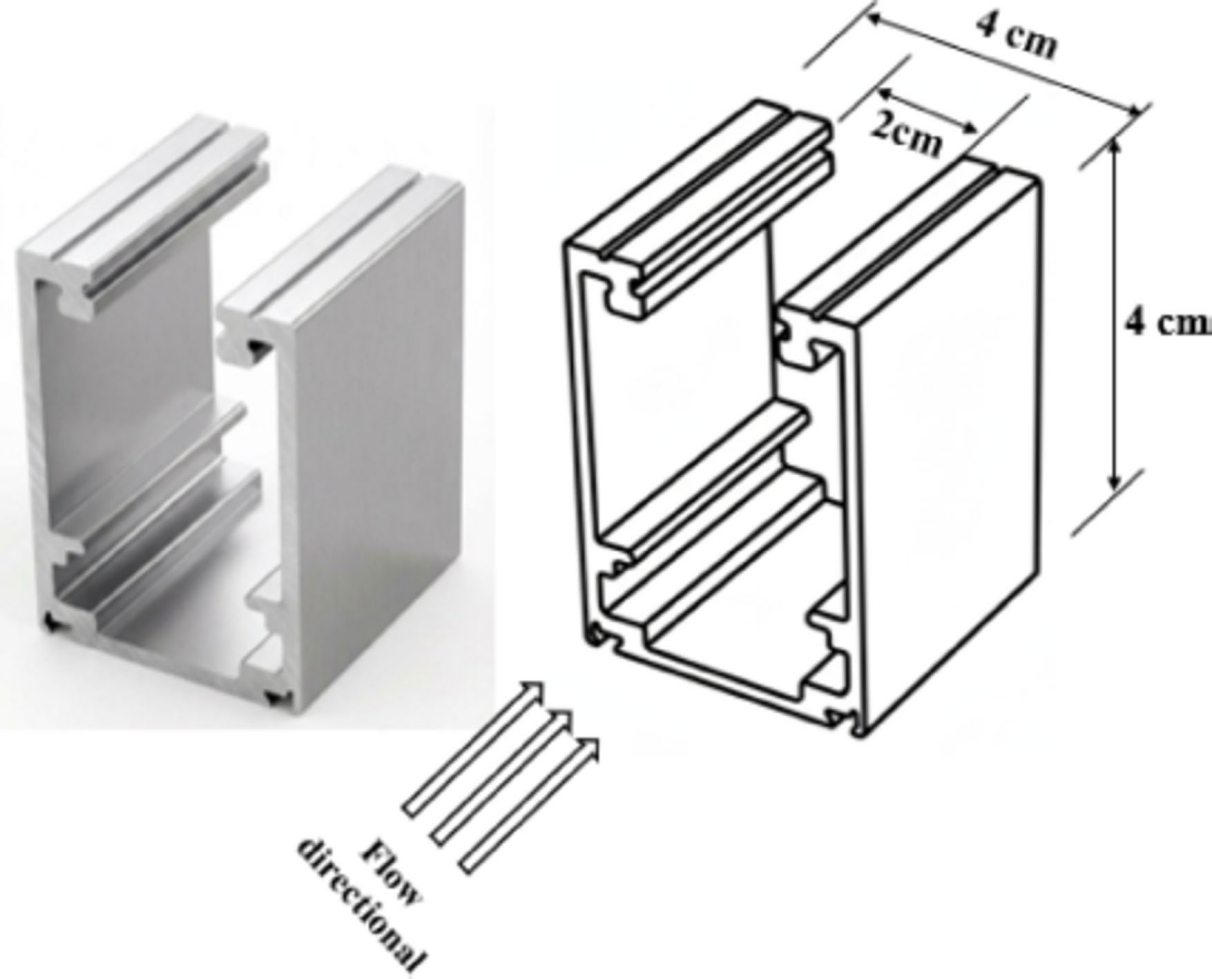
Photo of recycled aluminum interlocked channel fins.

**Fig. 4:**
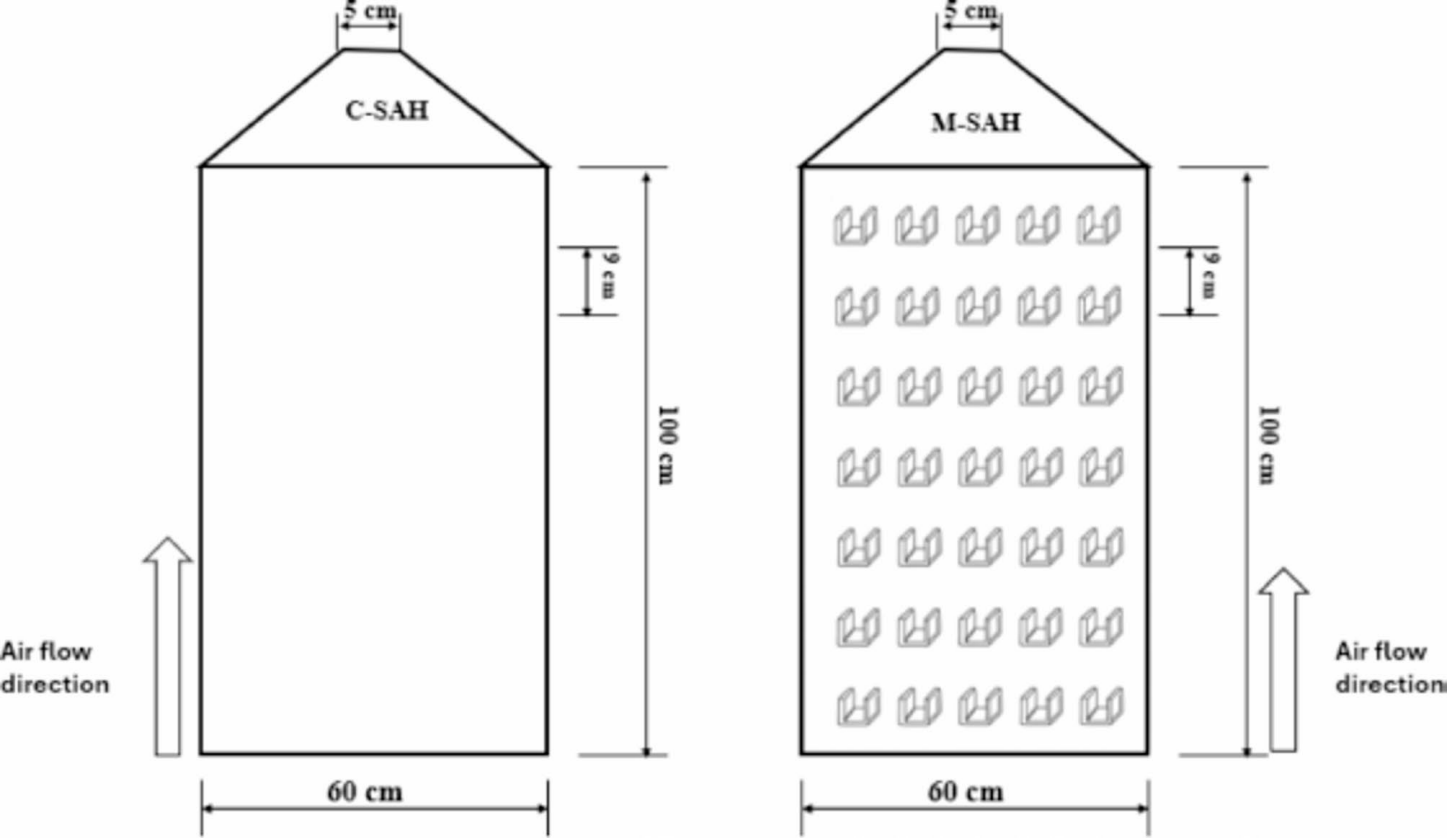
Arrangement of 35 recycled ICFs on the absorber plate in a 5-column × 7-row configuration. This assembly uses the geometry fin presented in Fig. 2 and photog in Fig. 3.

In the modified solar air heater (M-SAH), a novel interlocked channel fin (ICF), as shown in Fig. 3, was uniformly spaced and attached to the absorber surface. These fins consist of two modular extruded aluminum channels that mechanically interlock to form a confined airflow passage with integrated internal ribs. This configuration generates a structured flow path that improves the effective heat transfer area while simultaneously increasing turbulence within the air stream. The fins are made from recycled aluminum sections with a wall thickness of 1.5 mm. Each U-shaped fin has a length of 4 cm, a width of 4 cm, and a height of 4 cm. About 35 fin modules were arranged in a 7-row by 5-column matrix through the 100 × 60 cm absorber plate, resulting in a longitudinal pitch of 14.3 cm and a transverse pitch of 12.0 cm. The fin height of 4.0 cm within the 10 cm high duct leads to a flow blockage ratio of 13.3%, optimally balancing turbulence generation with pressure drop penalties. The use of the 35 fins and their internal ribs raises the total effective heat transfer area from the absorber area of 0.60 m² to approximately 0.92 m², producing a surface area improvement ratio of 1.5. The interlocking shape induces secondary flow structures and disrupts the thermal boundary layer, which improves the convective heat transfer coefficient. Furthermore, the modular assembly enhances structural rigidity, achieving uniform heat conduction from the absorber surface. Because of their low density, high thermal conductivity (~ 205 W/m·K), and environmental sustainability, the recycled aluminum profiles were chosen. The fins were surface polished to guarantee optimal contact with the absorber plate after being cut to the necessary proportions. To reduce the resistance to heat contact, they were mechanically secured. This successfully increased the absorber’s thermal surface and encouraged a more even diffusion of heat along the airflow. As seen in Fig. 4, about 35 fins were employed, grouped in 5 columns and 7 rows. Two 12 V DC axial fans with variable speed control were used to regulate the airflow at each unit’s inlet. This arrangement made it possible to operate at mass flow rates of 0.0046, 0.008, and 0.012 kg/s under both forced and natural convection. To assess the impact of the airflow regime on thermal augmentation, the performance of both heaters was compared under forced convection and natural convection at the three designated mass flow rates. In order to ensure steady operation and prevent grid-induced variations throughout the data collection process, the fans were powered by a 40 W photovoltaic panel that was coupled to a charge controller and a 12 V battery.

### Instrumentation

Measurements were made every day from 8:00 a.m. to 5:00 p.m. under the same weather conditions in order to assess the thermal performance of the modified solar air heater (M-SAH) and the conventional solar air heater (C-SAH). A digital pyranometer (SPM 72) was used to record solar radiation. A digital hot-wire anemometer (Testo 425) was utilized to measure the inlet air velocity for each heater. This instrument was chosen because it has acceptable precision at low air velocities inside ducts, as given in Table 3. The pyranometer and anemometer were calibrated based on the manufacturer’s specifications and checked under stable operating conditions. Temperature measurements were carried out using calibrated K-type thermocouples. The thermocouples were calibrated against a reference digital thermometer before the experiments. The inlet and outlet air temperatures were recorded to determine the temperature rise across each heater, while absorber plate temperatures were measured at several distributed points to obtain a representative average value. Glass cover temperatures were also monitored to assess convective and radiative heat losses. To achieve repeatability of measurements and decrease random errors multiple measurements were performed under identical operating conditions. For experiments involving natural convection operation, the airflow rate was determined from the measured outlet air velocity created by buoyancy-induced flow. Regarding forced convection, a calibrated hot-wire anemometer was used to measure the inlet air velocity at several locations across the heater cross-section and then averaged to obtain the representative velocity. In two cases, the mass flow rate was determined from the measured average velocity, air density, and cross-sectional area of the duct.

### Uncertainty analysis

To verify the reliability of the obtained data, an uncertainty analysis based on the standard propagation method was performed. The total experimental uncertainty, which was determined based on the accuracy of all devices used in the current study and presented in Table 3 is described in Eq. 1^48,49^.

1 $$\:{W}_{R}=\sqrt{\sum\:_{i=1}^{n}(\frac{\partial\:R}{\partial\:{x}_{i}}}{w}_{i}{)}^{2}$$

Where $\:{W}_{R}$ is the total uncertainty of a calculated quantity *R* due to the uncertainties in the input variables, *w*_*i*_ represents the uncertainty of the measure variable *x*_*i*_. Based on this method the uncertainty of the main performance parameters is given as follows:

The uncertainty of useful heat gain (Qu) is given as:

2 $$\:\frac{{W}_{Qu}}{Qu}=\left[\right(\frac{{w}_{m}}{m}{)}^{2}+(\frac{{w}_{\varDelta\:T}}{\varDelta\:T}{)}^{2}{]}^{1/2}$$

The uncertainty of thermal efficiency is given as:

3 $$\:\frac{{W}_{\eta\:th}}{\eta\:th}=\left[\right(\frac{{w}_{Qu}}{Qu}{)}^{2}+(\frac{{w}_{A}}{A}{)}^{2}+(\frac{{w}_{I}}{I}{)}^{2}{]}^{1/2}$$

The uncertainty of exergy efficiency is given as

4 $$\:\frac{{W}_{\eta\:ex}}{\eta\:ex}=\left[\right(\frac{{{W}_{Ex}}_{out}}{Ex,out}{)}^{2}+(\frac{{{W}_{Ex,}}_{in}}{Ex,in}{)}^{2}{]}^{1/2}$$

By substituting the accuracy of instruments from Table 3, into the above equations, the relative uncertainty of thermal efficiency was ± 2.32% and for exergy efficiency was ± 2.54%.

**Table 3:** Specifications of measuring instruments used in this study.

Device		Range	Accuracy
Thermocouple	Type K	−50–1300 °C	± 0.1 °C
Solar meter	Digital pyranometer - SPM 72	0–1999 Wm^− 2^	± 10 Wm^− 2^
SAHs area	Measuring tape	0–3 m	± 2%
Anemometer	Testo 425-Vane Anemometer	0.2–20 ms^− 1^	± 0.5 ms^− 1^

## Theoretical analysis for SAHs

### Thermal and exergy analysis for each SAH

The obtained useful heat gain $\:{Q}_{u}$ for the air passing through each solar air heater is calculated by^30^:

5 $$\:{Q}_{u}={\dot{m}}_{a}{C}_{p}\left({T}_{a,\:out}-{T}_{a,\:inlet}\right)$$

where $\:{T}_{a,\:out}$, $\:{T}_{a,\:inlet}$ were air temperature at outlet and inlet, respectively, $\:{\dot{m}}_{a}$ was the mass air flow rate Kg/s is obtained as:

6 $$\:{\dot{m}}_{a}={\rho\:}_{a}A{V}_{a}$$

where, Cp and $\:{\rho\:}_{a}$ were specific heat of air and density at average temperature $\:Tav=({T}_{a,\:out}+{T}_{a,\:inlet})/2$, they obtained as Eqs. 4 and 5^50,51^

7 $$\:{\rho\:}_{a}\left(Tav\right)=4.14068-0.01971\times\:Tav+4.9369\times\:{10}^{-5}\:\times\:{Tav}^{2}-7.04254{\times\:10}^{-8}\times\:\:{Tav}^{3}+5.74168{\times\:10}^{-11}\times\:\:{Tav}^{4}$$

8 $$\:{C}_{p}\left(Tav\right)=1052.350175-0.34816\times\:Tav+6.2735\times\:{10}^{-4}\:\times\:{Tav}^{2}+3.17305\times\:{10}^{-7}\times\:\:{Tav}^{3}-9.78603{\times\:10}^{-10}\times\:\:{Tav}^{4}$$

The amount of energy input is the received inclined solar radiation, which is absorbed by the SAH absorber. The absorber heat (Q_abs_) can be obtained as^52,53^:

9 $$\:\mathrm{Q}\mathrm{a}\mathrm{b}\mathrm{s}={\upalpha\:}\:{\uptau\:}\:\mathrm{A}\:\mathrm{I}$$

where τ is the glass transmittivity (0.96), α is the absorber plate absorptivity (0.95), and I is the solar radiation intensity (W/m^2^).

The absorbed thermal efficiency was calculated as the ratio between the useful energy gain and the absorbed heat by absorber plate^52,53^.:

10 $$\:{\eta\:}_{th}=\frac{{Q}_{u}}{Qabs}=\frac{{\dot{m}}_{a}{C}_{p}\left({T}_{a,\:out}-{T}_{a,\:inlet}\right)}{{\upalpha\:}\:{\uptau\:}\:\mathrm{A}\:\mathrm{I}}$$

The use of this efficiency to specifically estimate the thermal use and internal heat transfer characteristics of modified SAH has been performed and validated in previous studies such as^52–55^.

To achieve the best possible use of energy for any heating system, applying exergy analysis to plan the performance of each SAH is essential. The equations for exergy per unit time when considering a solar air heater steady system can be expressed as follows^56,57^:

11 $$\:\sum\:{\dot{E}x}_{in}-\:\sum\:{\dot{E}x}_{out}=\sum\:{\dot{E}x}_{dest}$$

where $\:{\dot{E}x}_{in}$, $\:{\dot{E}x}_{out}$, and $\:{\dot{E}x}_{dest}$ are the exergy input and output, and destruction, respectively. And input exergy is the exergy of the sun and the calculated $\:{\dot{E}x}_{out}$ is exergy gained by the air in solar air heater is obtained as:

12 $$\:\dot{E}{x}_{in}=I\left(1-\frac{4{T}_{amb}}{3{T}_{sun}}+\frac{1}{3}{\left(\frac{{T}_{amb}}{{T}_{sun}}\right)}^{4}\right)$$

13 $$\:\dot{E}{x}_{out}={\dot{m}}_{a}{C}_{p}\left[\left({T}_{a,\:out}-{T}_{amb}\right)-{T}_{o}\mathrm{ln}\frac{{T}_{a,\:out}}{{T}_{amb}}\right]$$

Where, Tsun is the sun surface temperature (as a black body Tsun ≅ 5777 K)^58^, $\:{T}_{amb}$ is the ambient temperature in the experimental area, The exergy efficiencies are given as the ratio of the absorbed exergy to the heating air to total input exergy to the SAH:

14 $$\:{\eta\:}_{ex}=\frac{\dot{E}{x}_{out}}{\dot{E}{x}_{in}}$$

### Economic analysis

An economic analysis was performed to assess the financial viability of the conventional and modified solar air heaters. To ensure the reliability of the economic model, specific assumptions were proposed based on the local economic context. The analysis included estimating the capital cost, operating cost, maintenance cost, and the financial savings resulting from reduced electricity consumption. The initial investment cost for the solar air heater system is primarily based on the cost of the recycled finned absorber panel, the glass cover, the insulation layer, the frame, and the small DC fans used for forced convection. The system lifespan (n) was set to 10 years, which accounts for the degradation of the glass cover, wooden casing, and DC fans. The annual interest rate (i) was set at 12%, indicating recent local commercial lending rates. The annual maintenance cost was evaluated at 15% of the initial capital. The salvage value was taken as 20% of CAT, which indicates the scrap value of the glass and aluminum fins at the end of the life of the SAH. The detailed economic equations, including the Capital Recovery Factor (CRF), Sinking Fund Factor (SFF), and Total Annual Cost (TAC), are summarized in Table 4^59–61^.

**Table 4:** Summary and description of the economic assessment equations for SAHs^62^.

Parameter	Obtained by		Eq.
The Capital recovery coefficient (CRC)	$$\:\mathrm{C}\mathrm{R}\mathrm{C}=\frac{\mathrm{i}\:{(1+\mathrm{i})}^{\mathrm{n}}}{{(1+\mathrm{i})}^{\mathrm{n}}-1}$$	where $$\:\mathrm{i}$$ is the yearly interest rate (12%), and n is the lifetime of SAHs (10 years)	(15)
The first year obtained (CFY)	$$\:\mathrm{C}\mathrm{F}\mathrm{Y}=\:\mathrm{C}\mathrm{R}\mathrm{C}\times\:\mathrm{C}\mathrm{A}\mathrm{T}$$	CAT is initial capital cost	(16)
The Sinking fund factor (SFF)	$$\:\mathrm{S}\mathrm{F}\mathrm{F}=\:\frac{\mathrm{i}\:}{{(1+\mathrm{i})}^{\mathrm{n}}-1}$$		(17)
The annual salvage cost (ASC)	$$\:\mathrm{A}\mathrm{S}\mathrm{C}=\mathrm{S}\mathrm{V}\times\:\mathrm{S}\mathrm{F}\mathrm{F}$$	$$\:\mathrm{S}\mathrm{V}$$ is the salvage value, taking as 20%P	(18)
The annual maintenance cost (ACM)	$$\:\mathrm{A}\mathrm{C}\mathrm{M}=\:15\mathrm{\%}\:\:\mathrm{C}\mathrm{A}\mathrm{T}$$		(19)
The total annual cost	$$\:\mathrm{A}\mathrm{C}\mathrm{T}=\mathrm{C}\mathrm{F}\mathrm{Y}+\mathrm{A}\mathrm{C}\mathrm{M}-\mathrm{A}\mathrm{S}\mathrm{C}$$		(20)
Energy cost ($/kWh)	$$\:\mathrm{C}\mathrm{P}=\frac{\mathrm{A}\mathrm{C}\mathrm{T}}{\mathrm{A}\mathrm{n}\mathrm{n}\mathrm{u}\mathrm{a}\mathrm{l}\:\mathrm{u}\mathrm{s}\mathrm{e}\mathrm{f}\mathrm{u}\mathrm{l}\:\mathrm{e}\mathrm{n}\mathrm{e}\mathrm{r}\mathrm{g}\mathrm{y}}$$		(21)

### Energy metric

The EPBT indicates the time required for the solar air heater to recover the embodied energy associated with its construction through useful energy^63^.

22 $$\:{\mathrm{E}\mathrm{P}\mathrm{B}\mathrm{T}}_{\mathrm{e}\mathrm{n}}\:=\frac{{\mathrm{E}}_{\mathrm{e}\mathrm{m}}}{\:{{(\mathrm{E}}_{\mathrm{o}\mathrm{u}\mathrm{t}})}_{annual}}\:\:\:\left(\mathrm{y}\mathrm{e}\mathrm{a}\mathrm{r}\right)\:\:\:\:\:\:$$

23 $$\:{\mathrm{E}\mathrm{P}\mathrm{B}\mathrm{T}}_{\mathrm{e}\mathrm{x}}\:=\frac{{\mathrm{E}}_{\mathrm{e}\mathrm{m}}}{\:{\dot{{(\mathrm{E}\mathrm{x}}_{o})}}_{annual}\:}\left(\mathrm{y}\mathrm{e}\mathrm{a}\mathrm{r}\right)$$

### Exergo-economic analysis

The exergo-economics parameters ($\:{R}_{ex}$) for conventional and modified integrated solar air heater (M-SAH). The $\:{R}_{ex}$ used to express maximizing the relationship between thermal and economic performance^64,65^.

24 $$\:{R}_{ex}=\frac{{\dot{(E}{x}_{out})}_{annual}}{\mathrm{A}\mathrm{C}\mathrm{T}}$$

### Environmental impact analysis

Since the solar air collector system is a source of renewable energy, it aims to reduce carbon dioxide (CO2) emissions, which pose a major threat to the environment as they are the main cause of global warming. Considering the amount of energy used to heat the air with an electrical energy source and replacing this with solar energy reduces the emitted carbon dioxide, which has a negative effect on the environment. In addition, any improvement in the performance of the solar air collector will consequently lead to a reduction in the amount of these emissions throughout the period of operation of the solar collector. The mitigation of CO_2_ by ton over time ($\:{\theta\:}_{CO2\:}$) is obtained by^65,66^.

25 $$\:{\theta\:}_{CO2\:}=\frac{2\left({E}_{out-An}\times\:n\right)}{1000}\:\:\:\:ton$$

Where *E*_*out-An*_ is the annual output energy of SAH and *n* is its lifetime (10 years).

Carbon credit earned $$\:\mathrm{C}\mathrm{C}\mathrm{E}$$ used to predicate benefit of solar air heaters as income cash when considering the value of a ton of carbon dioxide saving in carbon markets $$\:\mathrm{\$}{CO}_{2}$$=14.5 $/ton CO_2_^65,67^.

26 $$\:\mathrm{C}\mathrm{C}\mathrm{E}=\mathrm{\$}{CO}_{2}\times\:{\theta\:}_{CO2\:}\:\:\:\mathrm{\$}$$

## The AI model

**Fig. 5:**
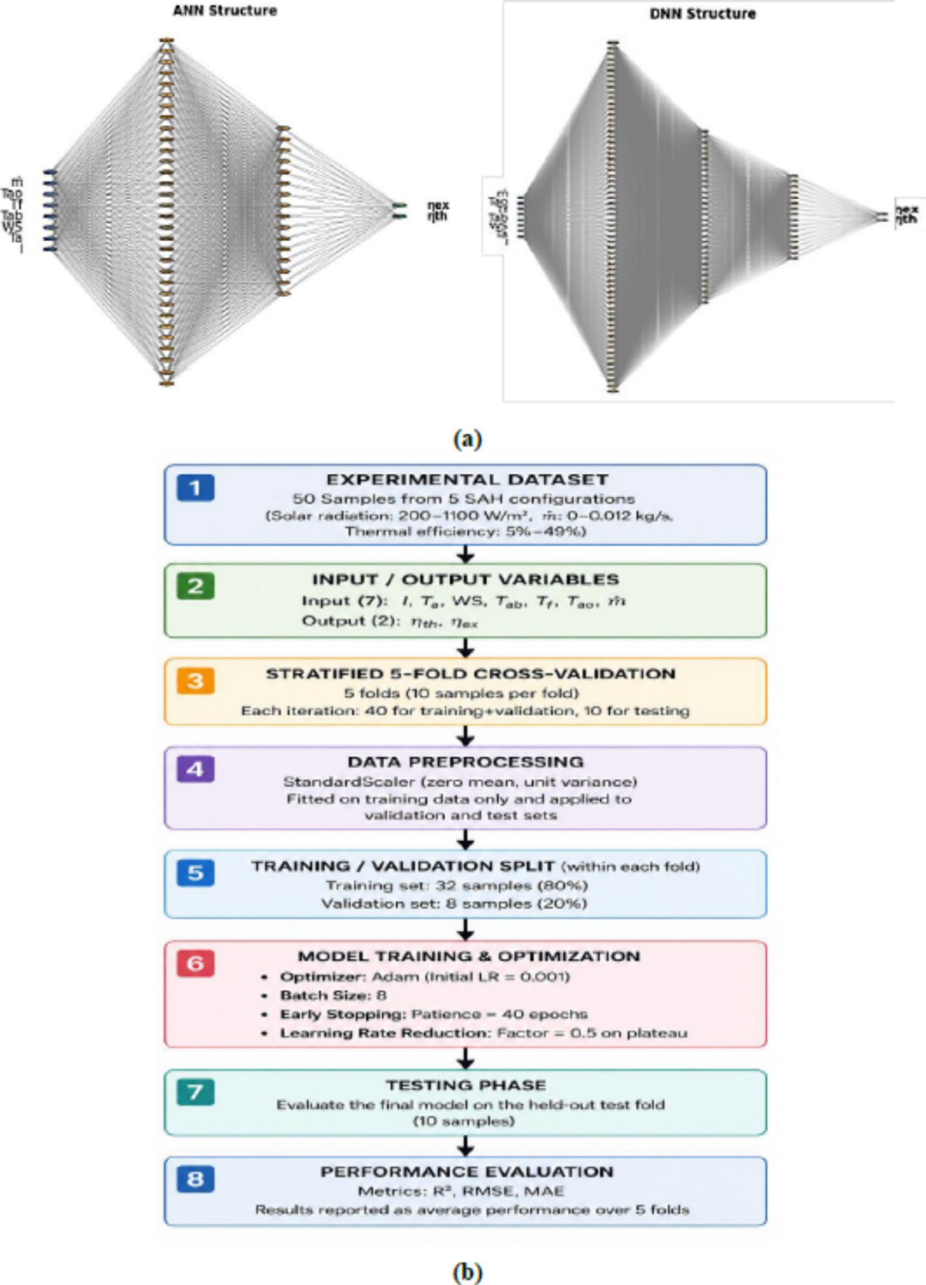
(**a**) The structure and (**b**) settings of models.

In this study, two AI models, deep neural network (DNN) and artificial neural network (ANN), were developed to predict the thermal and exergy efficiencies of solar air heaters at different designs. The choice of the architecture of the network depends on the nature of the underlying physical phenomenon. Solar air heater systems contain highly coupled phenomena, involving convective heat transfer, the absorption of solar energy, airflow dynamics, and thermal losses, which often lead to nonlinear behavior under different operating conditions. Therefore, the DNN model is more appropriate and necessary for these systems due to its ability to capture the complex nonlinear dynamics, such as forced-flow heat transfer, as a result of the added depth to capture intricate variable interactions. On the other hand, simpler models such as the ANN model are more suitable for systems involving quasi-linear behavior like exergy generation because they provide superior accuracy and stability while preventing the possible risk of overfitting associated with unnecessary model complexity. Models were developed using 50 datasets representing the experimental results of five configurations, including C-SAH, M-SAH, M-SAH0.0046, M-SAH0.008, and M-SAH0.012 at mass flow rates of 0.0046, 0.008, and 0.012 kg/s. The architectural structures of the two models were carefully selected because of the lower size of the datasets. The two models consist of an input layer, a number of hidden layers, and an output layer. The input layer contains 7 neurons representing input variables, mainly solar radiation (I), ambient air temperature (Ta), wind speed (WS), absorber plate temperature (Tab), fins temperature (Tf), outlet air temperature (Tao), and mass flow rates (m). The output layer is composed of two neurons representing energy and exergy efficiencies as target outputs. Before scaling and training the model, the dataset was rigorously screened. The absence of missing data points was achieved through continuous monitoring. Physical constraint checks, such as thermodynamic limits, were used to identify the outliers, while the Interquartile Range (IQR) statistical method was used to ensure the reliability of the training data. The ANN model was composed of two hidden layers with 32 and 16 neurons, a dropout of 0.25, a ReLU activation function, and batch normalization. The DNN models had 3 hidden layers, with a structure of 64→32→16, and a higher dropout of 0.35 to improve generalization. An iterative, constrained hyperparameter optimization was carried out to avoid suboptimal parameter selection and to ensure a fair comparison. With the limited size of the dataset, exhaustive automated searches, such as large-scale Grid Search, were intentionally avoided to prevent overfitting the validation folds. Instead, models were systematically evaluated across a limited, theoretically justified range (1–3 hidden layers, 8–64 neurons, dropout 0.1–0.5). The final architectures of ANN and DNN models were selected as they consistently achieved the lowest validation loss across the 5-fold cross-validation without overfitting. This demonstrates that the superior performance of the ANN for parameters such as exergy efficiency is a result of its optimal match for the quasi-linear data, instead of suboptimal tuning. The Adam algorithm was used to train the models with a small batch size of 8 samples, to fit a lower dataset, and a learning rate of 0.001 to improve convergence. An automatic decrease in the learning rate at fixed loss and early stopping, with a tolerance of 40 cycles, were conducted. A 5-fold cross-validation technique was used to avoid data leakage and ensure robust model assessment. The dataset was stratified based on mass flow rate categories to maintain representative distributions across all folds. In each iteration, the data was split into 20% for testing (10 samples) and 80% for training/validation (40 samples). Furthermore, within the training set, a further split of 80/20 was used for actual validation (8 samples) and training (32 samples) to avoid overfitting. A StandardScaler was used to normalize all input features, fitted exclusively on the training data of each fold and then applied to the validation and test sets. Early stopping with an automatic learning rate reduction and a patience of 40 epochs was performed to improve convergence and avoid overfitting. The model performance was evaluated using different metrics such as the coefficient of determination (R²), root mean squared error (RMSE), and absolute mean error (MAE), in addition to the cross-validation technique. Figure 5 shows the structure and settings of the two models.

## Results and discussions

### Measurements of the Atmosphere

**Fig. 6:**
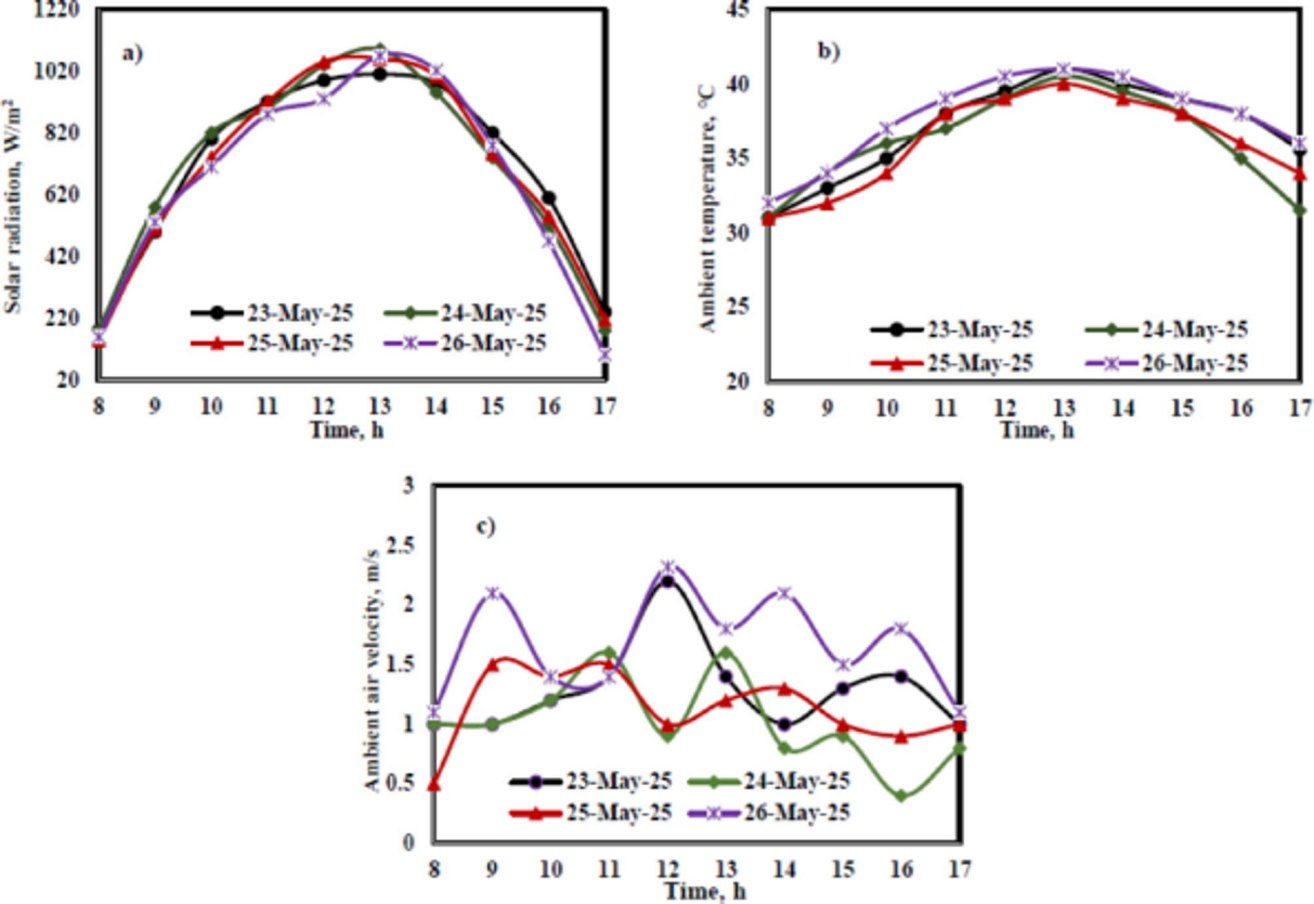
Fluctuations of sun intensity, ambient temperatures wind speed.

The hourly changes in the ambient atmospheric conditions across the four experimental days between 8:00 and 17:00 are depicted in Fig. 6(a-c). Figure 6a illustrates how solar radiation steadily rises from the early morning until it peaks at 13:00, with peak values ranging from 1010 to 1090 W/m². After that, it gradually falls until sunset. The experiments were carried out under almost the same solar radiation setup, as evidenced by the same daily trends and convergence of peak values. At 8:00, the ambient temperature increases gradually from roughly 31 °C to peak values of 40–41 °C. Rather, as Fig. 6c illustrates, the ambient wind speed varied over the course of the day ranged between 0.4 and 2.32 m/s, with similar trends observed on all experimental days. Typically, these wind speeds fall within the low to moderate range of 5 m/s^47,48^. Having considered everything, the weather supports the idea that these tests were carried out in a steady and reliable atmosphere.

### Solar air heater temperatures

#### Outlet air temperature

**Fig. 7:**
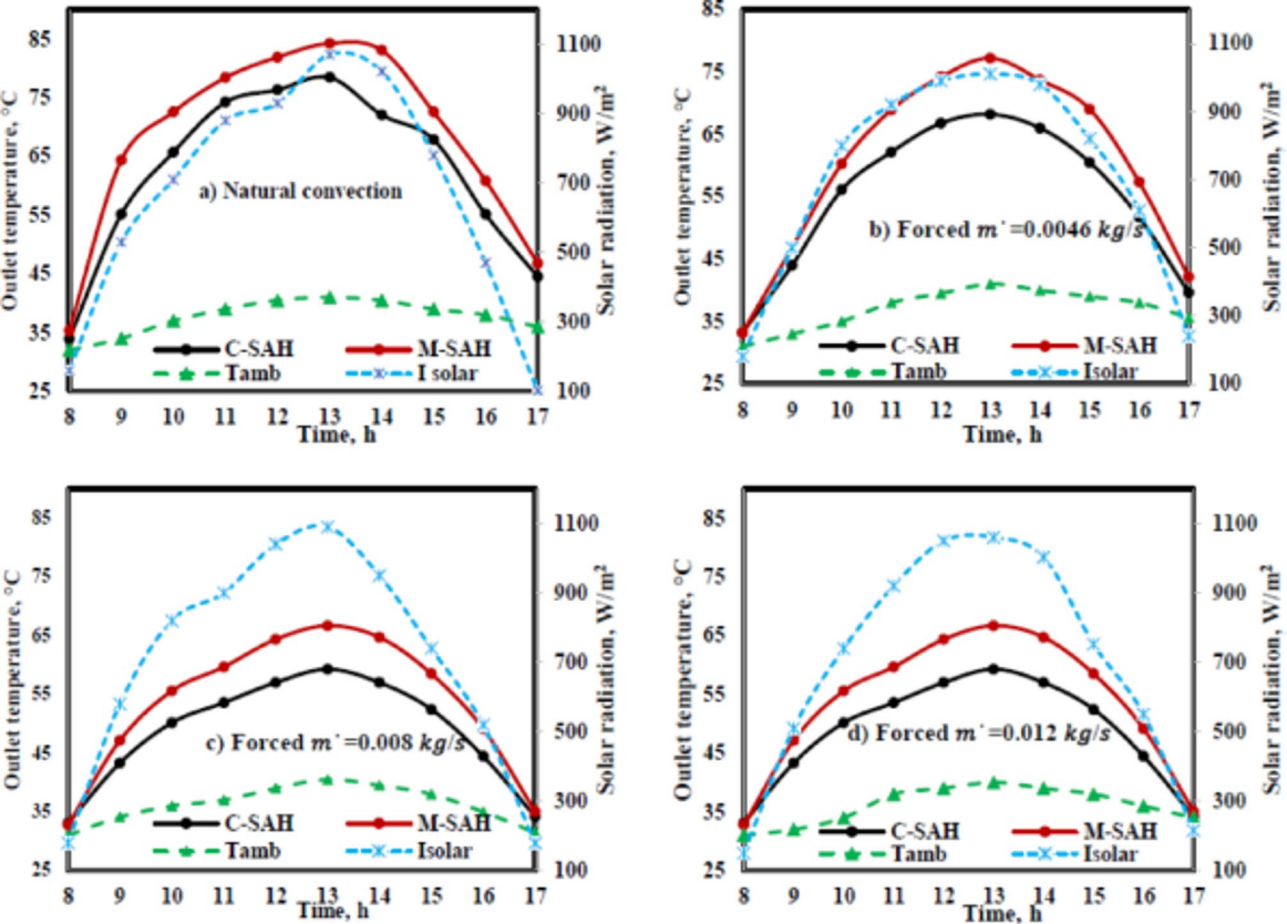
Comparing hourly outlet temperatures for conventional and modified SAH at (**a**) natural convection and at mass flow rates (**b**) 0.0046 kg/s, (**c**) 0.008 kg/s, (**d**) 0.012 kg/s.

Figure 7 presents the outlet air temperature behavior for the conventional SAH (C-SAH) and the modified SAH (M-SAH) operating under natural convection and forced convection modes at different mass flow rates (0.004, 0.008, and 0.012 kg/s). In general, both SAHs follow a similar daily trend: the outlet air temperature increases from early morning as solar radiation increases, reaches a peak at 13:00, and then decreases gradually toward sunset as solar intensity declines. Under natural convection conditions, as shown in 7a, the M-SAH provides a higher outlet temperatures are higher for the M-SAH than for the C-SAH over the entire operating period. The maximum outlet temperature reaches about 84 °C for the M-SAH. In comparison, it is about 78 °C for the C-SAH at 13:00. This improvement is mainly due to the use of recycled aluminum fins, which increase the effective heat transfer area and enhance heat exchange between the absorber surface and the airflow under buoyancy-driven conditions. Figure 7b illustrates the outlet air temperature profiles under forced convection at the lowest mass flow rate of 0.0046 kg/s. The M-SAH shows noticeably higher outlet temperatures throughout the day compared to the C-SAH. The peak outlet temperature of the M-SAH reaches about 77 °C, whereas the C-SAH records about 68 °C. At a mass flow rate of 0.008 kg/s, as shown in Fig. 5c, both SAHs exhibit lower maximum outlet temperatures than in the lower mass flow rate case. The C-SAH attains a peak temperature of about 59 °C, while the M-SAH reaches about 67 °C. For the highest tested mass flow rate of 0.012 kg/s, shown in Fig. 5d, a further decrease in outlet air temperature is observed for both systems. The maximum outlet temperature is about 54 °C for the C-SAH and about 60 °C for the M-SAH. In general, the M-SAH maintains a clear thermal advantage, indicating that the recycled aluminum fins partially compensate for the reduced contact time by sustaining a higher heat transfer rate between the absorber and the airflow.

**Fig. 8:**
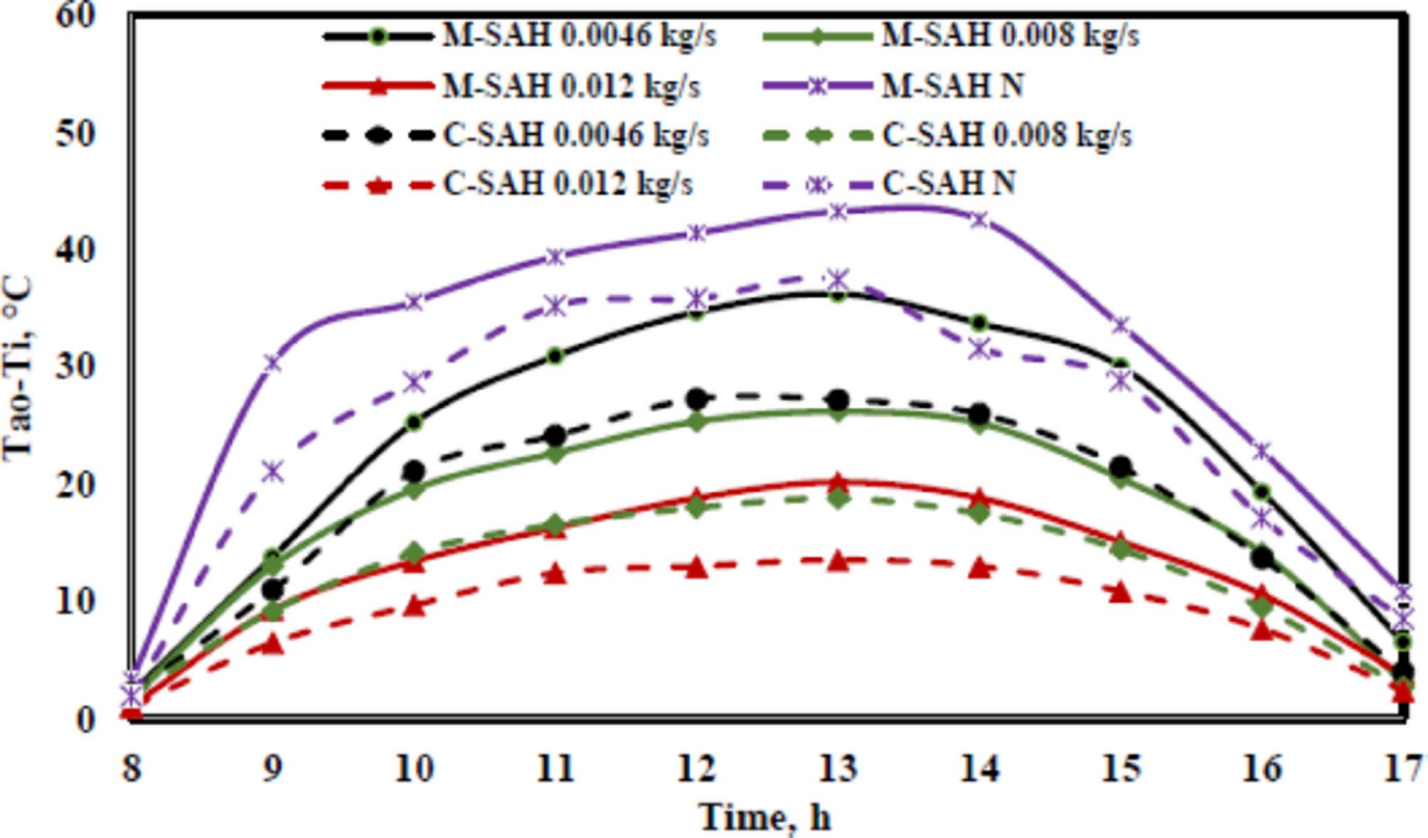
Variation of temperature difference for C-SAH and M-SAH under natural and forced convection at different mass flow rates 0.0046 kg/s, 0.008 kg/s, and 0.012 kg/s.

Figure 8 presents the hourly variation of the air temperature rise (ΔT) for both C-SAH and the M-SAH under natural convection and forced convection at mass flow rates of 0.0046, 0.008, and 0.012 kg/s. As shown in Fig. 6, the M-SAH consistently produces a higher temperature rise than the C-SAH during the entire operating period and for all flow regimes. Under natural convection, the temperature rise of the M-SAH increases from 3.3 °C at 08:00 to 43.2 °C at 13:00, whereas the C-SAH reaches 37.4 °C at the same time. This difference of 5.8 °C at peak conditions reflects the contribution of the recycled aluminum U-shaped fins, which increases the effective heat transfer area and improve the thermal interaction between the absorber plate and the airflow.

**Fig. 9:**
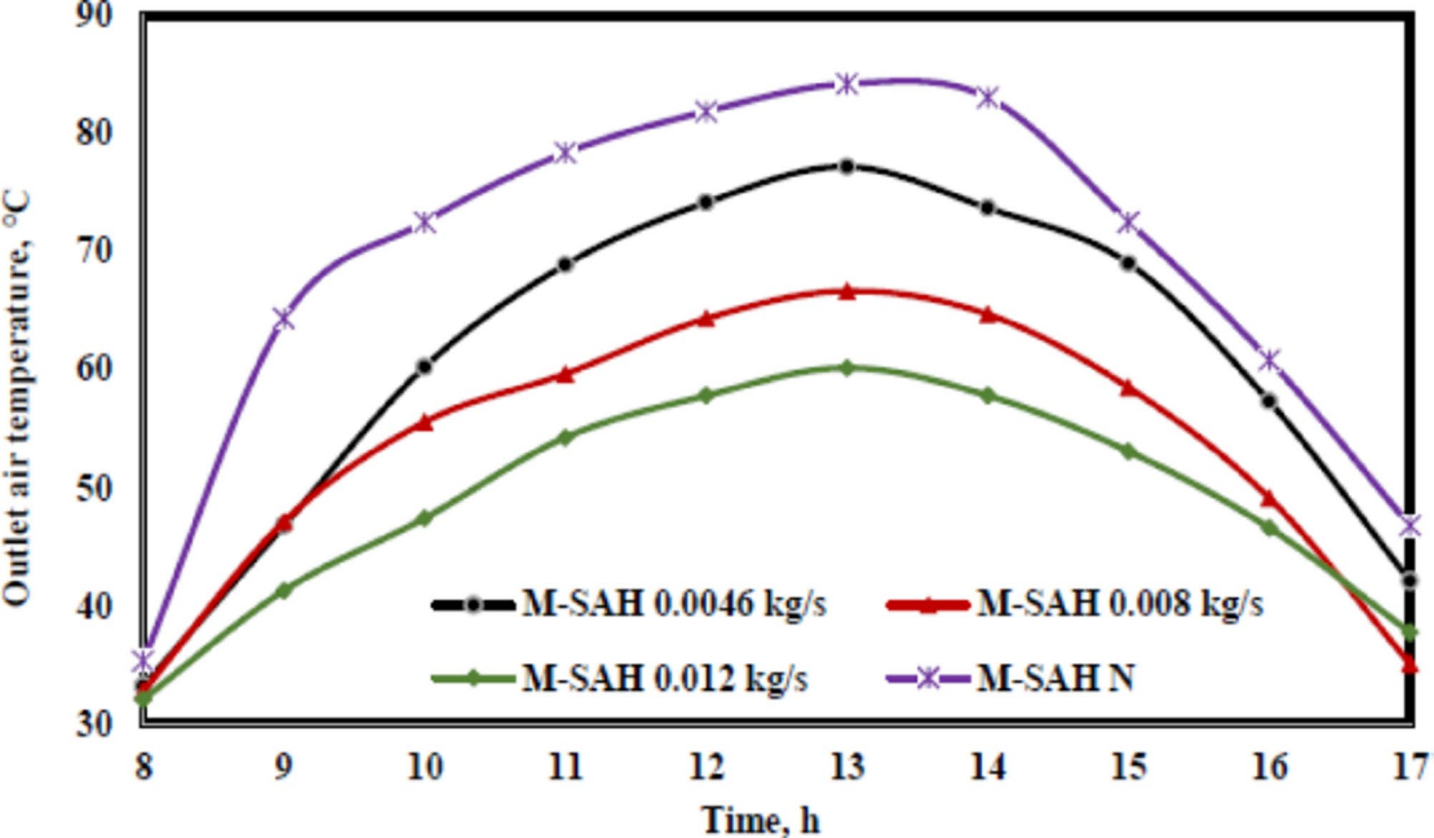
Comparison of outlet air temperature of the M-SAH under natural and forced convection at different mass flow rates 0.0046 kg/s, 0.008 kg/s, and 0.012 kg/s.

The same behavior is clearly observed in Fig. 9, where the outlet air temperature of the M-SAH under natural convection rises from 35 °C at 08:00 to 84 °C at 13:00, due to the longer air residence time inside the duct. Under forced convection, increasing the mass flow rate reduces the outlet temperature, where with the peak value decreasing from about 77 °C at 0.0046 kg/s to 67 °C at 0.008 kg/s and 60 °C at 0.012 kg/s. This trend is mainly attributed to the reduced residence time at increased flow rates, which limits the heat gain of the air despite the enhanced heat transfer provided by the recycled aluminum fins.

#### Absorber temperature

**Fig. 10:**
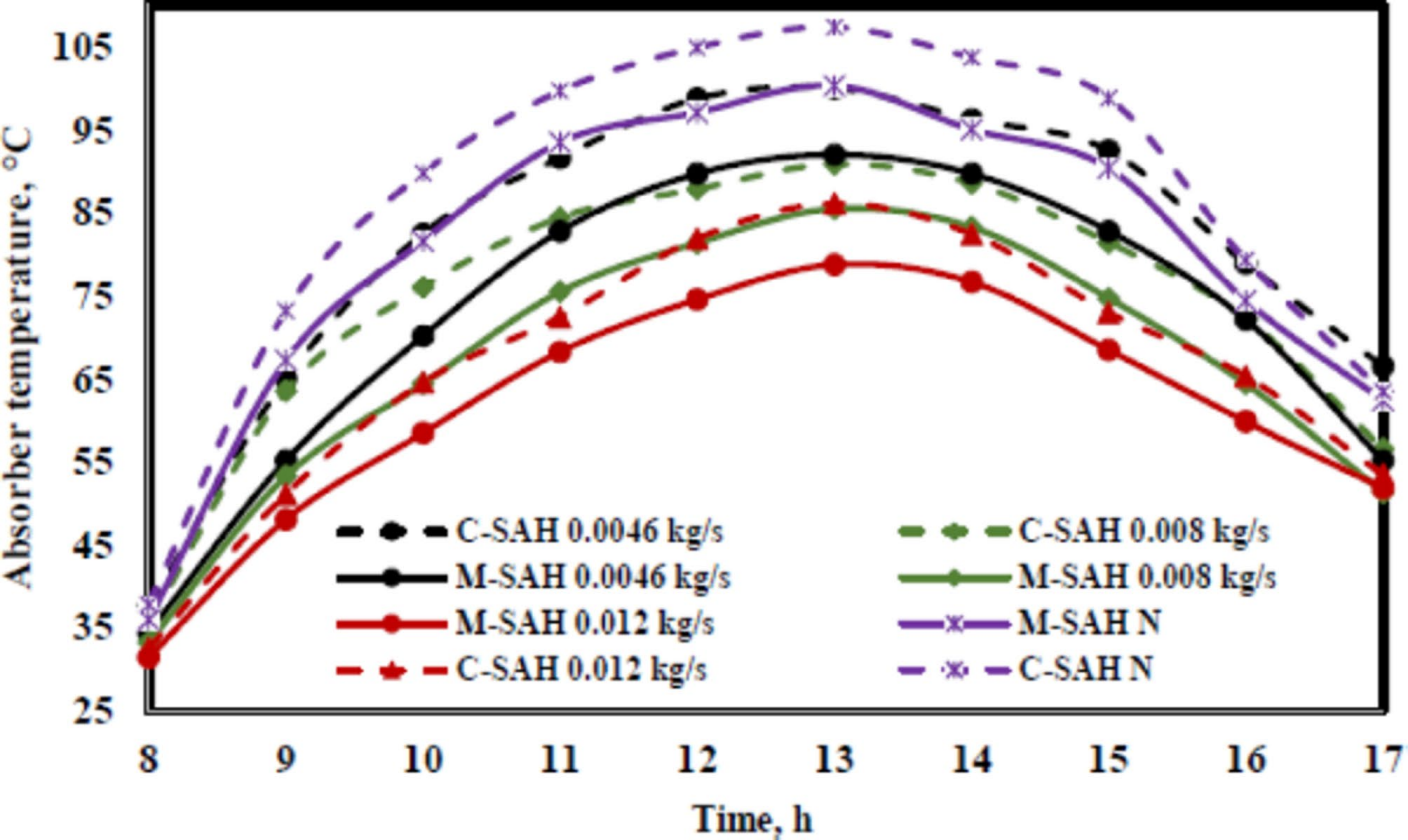
The hourly absorber temperature variation for each C-SAH and M-SAH under natural and forced convection at different mass flow rates 0.0046 kg/s, 0.008 kg/s, and 0.012 kg/s.

The hourly variation of the absorber temperature for each C-SAH and M-SAH under forced convection and natural convection at various mass flow rates throughout the test day is shown in Fig. 10. Under all working conditions, the absorber temperature rises from 8:00, peaks at 13:00, and then falls approaching 17:00 in response to variations in the intensity of solar light. The absorber temperature of the C-SAH rises to 38 °C at 08:00, peaking at 107 °C at 13:00 under natural convection, whereas the M-SAH achieves a 100 °C, indicating a decrease of more than 6.5%. This is because the recycled aluminum fins promote heat absorption. For both SAHs, raising the mass flow rate under forced convection results in a noticeable drop in absorber temperature. The absorber temperature of the C-SAH drops about 14% at 13:00, from roughly 100 °C at 0.0046 kg/s to 91 °C and 86 °C at 0.008 and 0.012 kg/s, respectively. A similar trend is observed for the M-SAH, where the absorber temperature decreases from 92 °C to 79 °C as the mass flow rate increases, giving a comparable reduction of nearly 14%. Throughout the test period, the M-SAH consistently exhibits lower absorber temperatures than the C-SAH under identical airflow conditions, with an average reduction in the range of 5–10%, confirming that the recycled aluminum fins enhance heat transfer by convection from the absorber surface to the airflow.

**Fig. 11:**
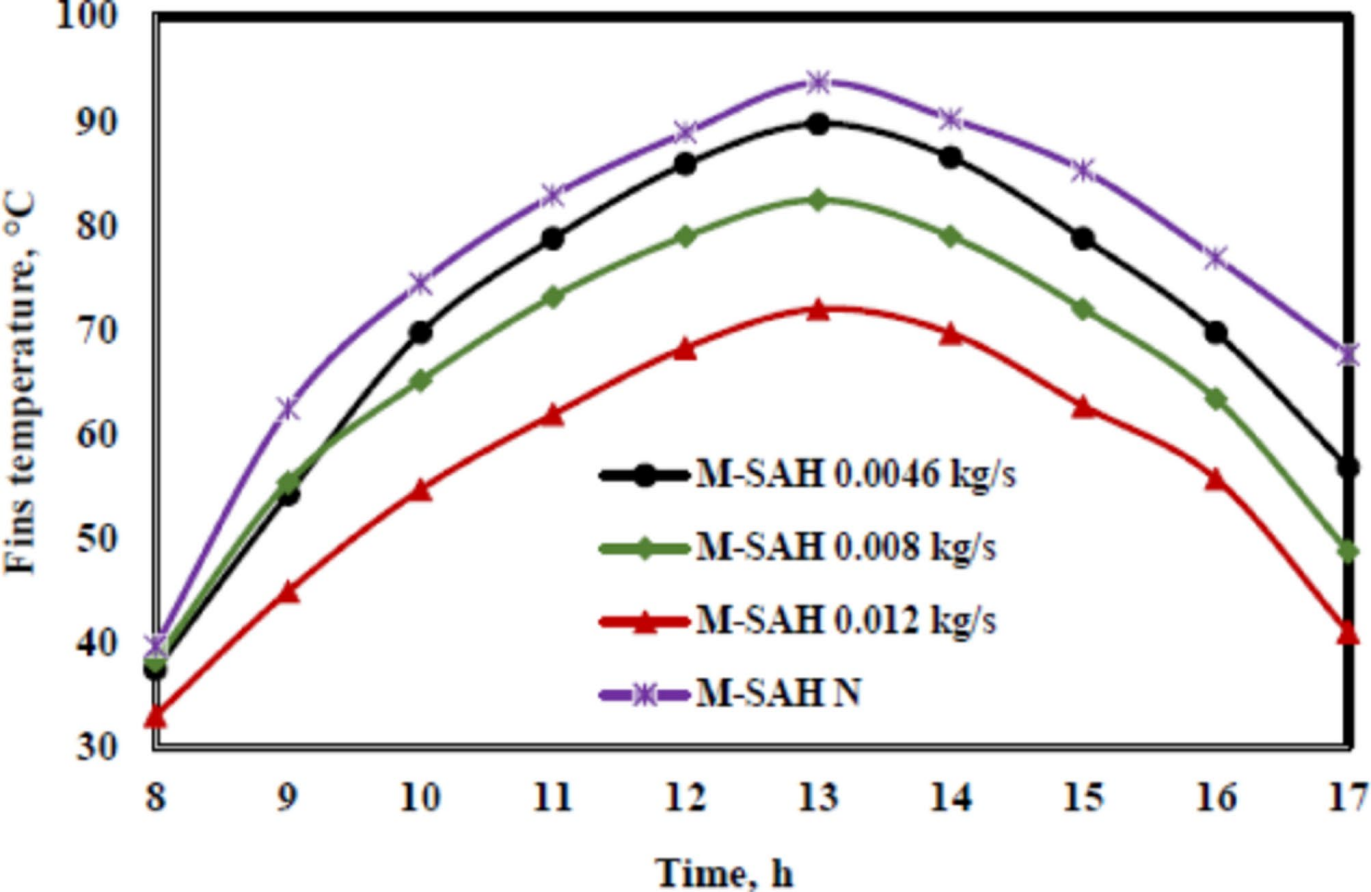
Hourly variation of fins temperature for forced at three mass flow rates 0.0046, 0.008, and 0.012 Kg/s.

The fin temperature exhibits a similar profile fluctuation to the absorber plate temperature behavior (see Fig. 11), peaking around 13:00 and then decreasing. With the induction of forced convection at mass flow rates of 0.0046, 0.008, and 0.012 kg/s, the maximum fin temperature drops to 90 °C, 82 °C, and 72 °C, respectively. In contrast, in the case of natural convection, it recorded the highest peak of 94 °C.

### Results of energy analysis

**Fig. 12:**
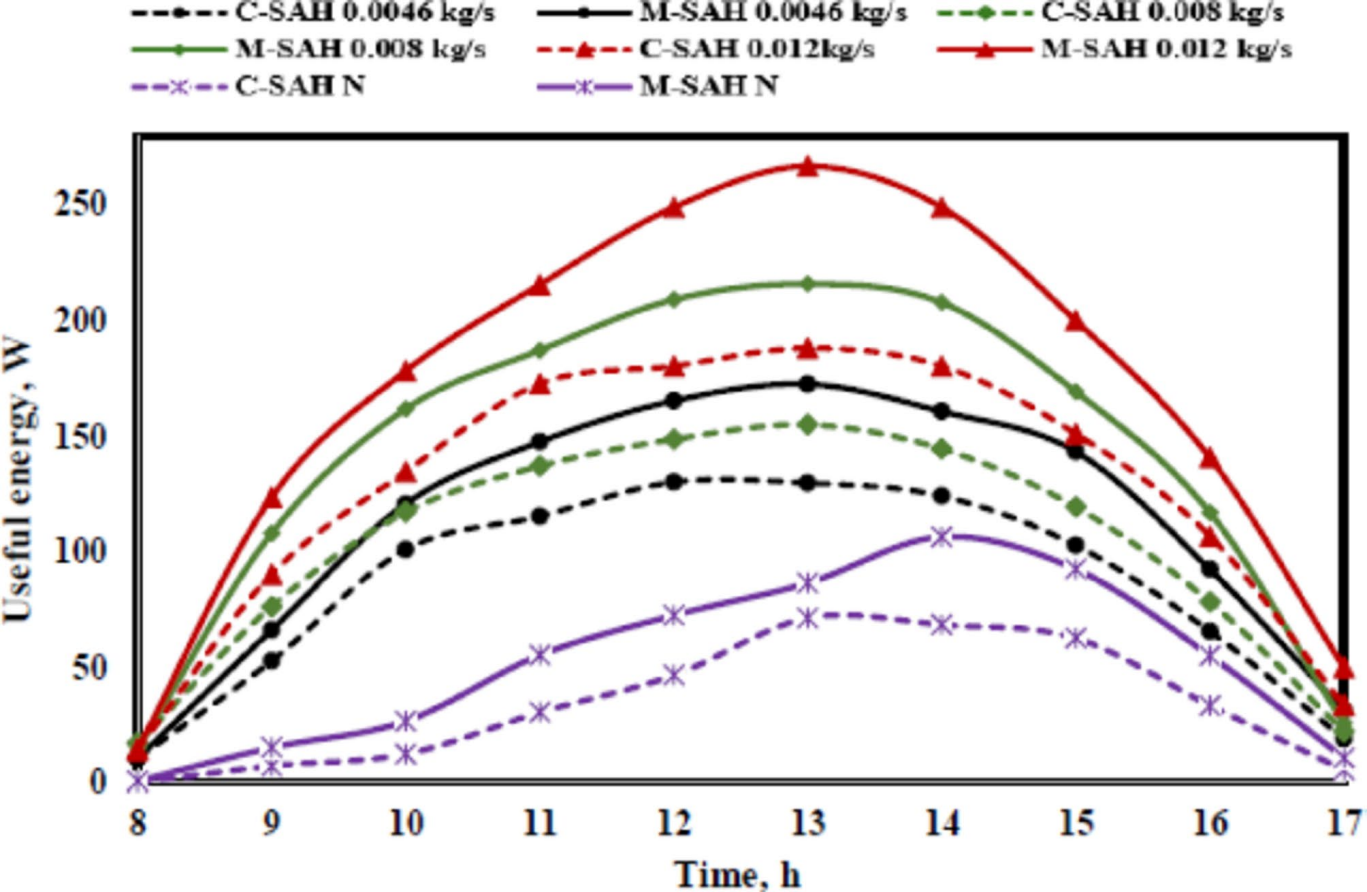
The hourly useful energy for C-SAH and M-SAH under natural and forced convection at different mass flow rates 0.0046 kg/s, 0.008 kg/s, and 0.012 kg/s.

The M-SAH continuously produces more useful energy than the C-SAH under all operating settings illustrated in Fig. 12, demonstrating the beneficial contribution of the incorporated recycled aluminum fins with the novel design to improve heat extraction. The M-SAH’s useful energy output under natural convection is 86 W at 13:00, while the C-SAH’s is 71 W. This shows a noticeable improvement even under flow circumstances driven by buoyancy. Under forced convection, where the impact of air mass flow rate is more noticeable, this enhancement becomes more pronounced. Peak useful energy output increases from 130 W for the C-SAH to 172 W for the M-SAH at a mass flow rate of 0.0046 kg/s, and from 155 W to 216 W at a mass flow rate of 0.008 kg/s. At the maximum tested mass flow rate of 0.012 kg/s, a similar pattern is shown, with the M-SAH producing a maximum usable energy output of 267 W, as opposed to 188 W for the C-SAH. Furthermore, both SAHs exhibit a significant increase in useable energy output when the air mass flow rate is increased, even while the outlet air temperature decreases.

**Fig. 13:**
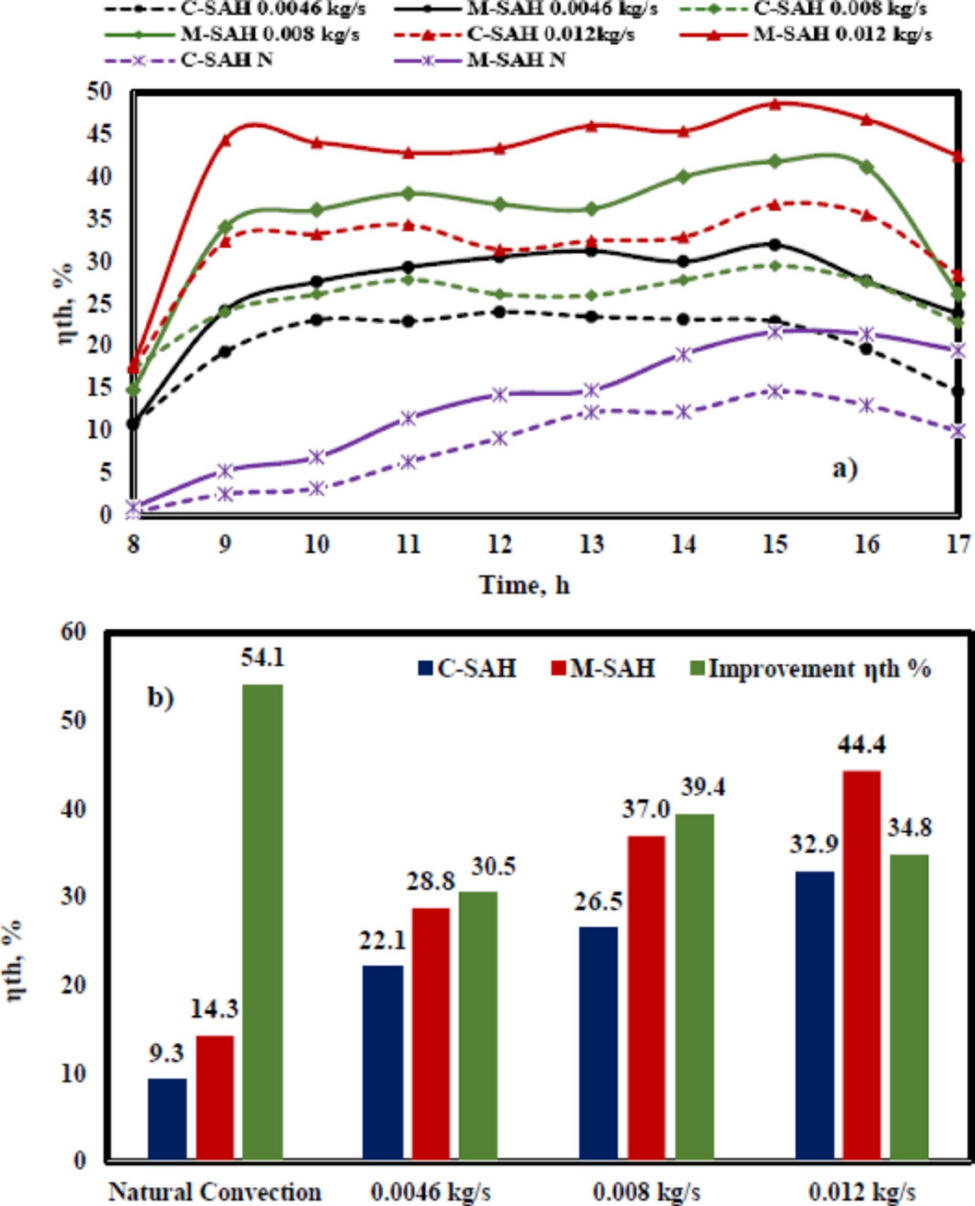
(**a**) hourly energy efficiency comparison and (**b**) average energy efficiency and improvements.

A solar air heater’s energy efficiency largely determines its thermal performance. The efficiencies of the modified solar air heater with recycled aluminum fins (M-SAH) and the conventional solar air heater (C-SAH) both rise in the morning, peak between midday and early afternoon, and then fall as the intensity of the sun’s rays decreases, as shown in Fig. 13a. Due to buoyancy constraints limiting airflow, both systems exhibit relatively poor efficiency under natural convection. With a peak efficiency of 21.7% at 15:00 compared to C-SAH’s 14.7%, M-SAH still has a distinct edge. The performance difference between the two systems is more noticeable when forced convection is used, underscoring the combined impact of fin-assisted heat transfer and airflow rate. With an air mass flow rate of 0.0046 kg/s, the M-SAH achieves a maximum efficiency of 31.2% at 13:00, while the peak thermal efficiency of the C-SAH is about 23.5%. However, at 15:00, the efficiencies are 31.9% and 22.9%, respectively. Performance is further improved by raising the mass flow rate to 0.008 kg/s; at 15:00, the M-SAH achieves 41.8% efficiency, while the C-SAH reaches 29.5%. The M-SAH achieves a peak thermal efficiency of 48.6% at the highest tested mass flow rate of 0.012 kg/s, while the C-SAH records a peak thermal efficiency of 36.7%. As observed on Fig. 13b, the M-SAH’s average daily performance also reflects its increased hourly efficiency. The average daily thermal efficiency under natural convection rises from 9.30% for the C-SAH to 14.33% for the M-SAH. With efficiencies increasing from 22.06% to 28.78% at 0.0046 kg/s, from 26.51% to 36.97% at 0.008 kg/s, and from 32.91% to 44.36% at 0.012 kg/s, under forced convection, significant improvements are shown at all airflow rates, translating into relative improvements of 30–39%. According to these findings, adding recycled aluminum fins improves overall energy conversion under all studied flow conditions by steadily increasing hourly and daily thermal efficiency.

### Results of exergy analysis

**Fig. 14:**
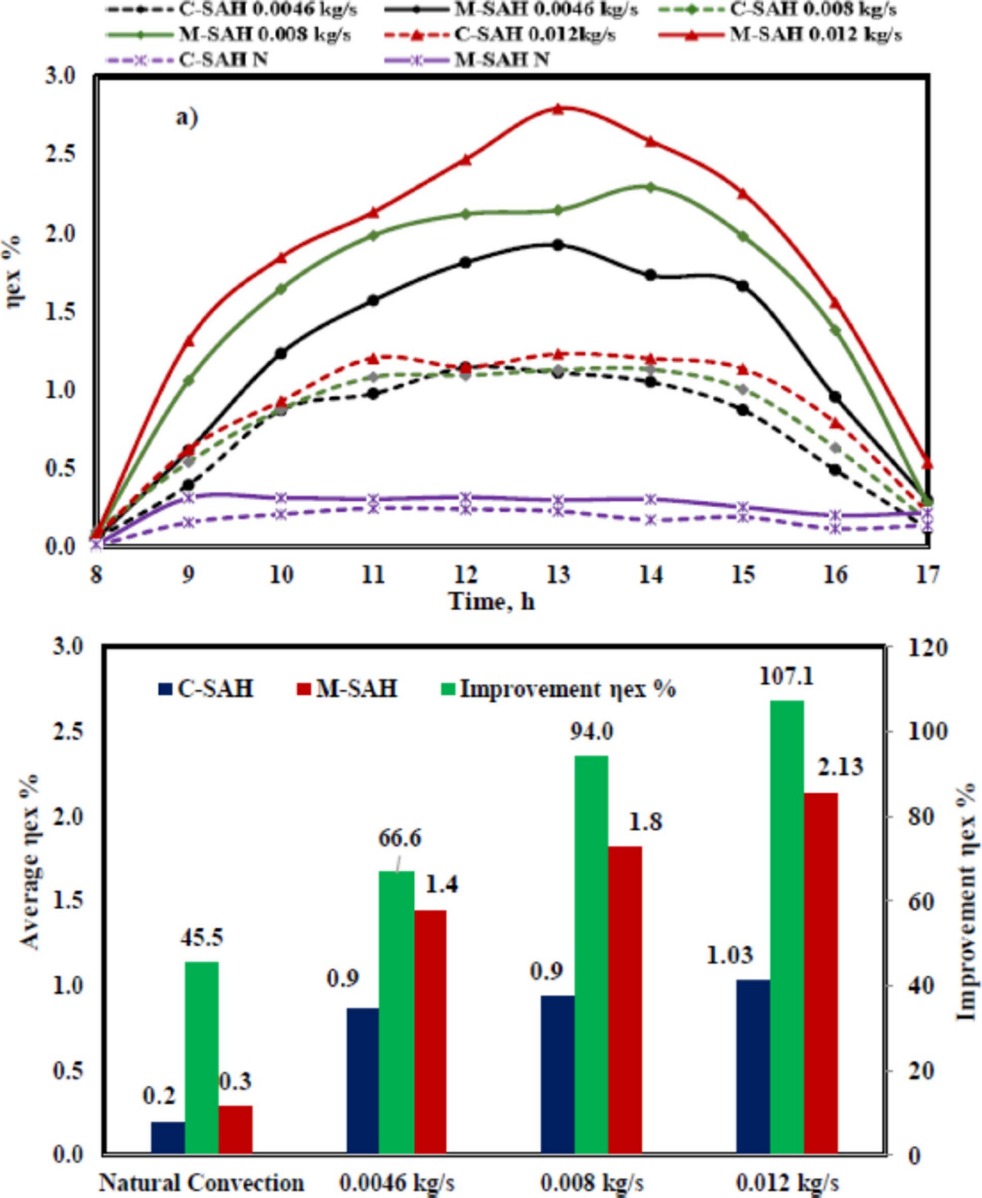
(**a**) hourly exergy efficiency comparison and (**b**) average exergy efficiency and improvements compared each case with its C-SAH.

The solar air heaters’ capability for converting absorbed solar energy into high-quality, usable energy is demonstrated by their hourly exergy efficiency, which is displayed in Fig. 14a. Because of the restricted air mass flow caused by buoyancy forces, efficiency under natural convection remains very low. The M-SAH, however, continuously performs better than the C-SAH, reaching a high energy efficiency of 0.30% as opposed to 0.24% for the traditional heater. The performance gap between the systems is further widened by forced convection. At 11:00, the M-SAH surpasses the C-SAH value of 0.97% by reaching 1.57% at 0.0046 kg/s. The M-SAH reaches 2.80% at 0.012 kg/s, surpassing 1.23% for the C-SAH, and peaks at 2.29% at 0.008 kg/s, compared to 1.13% for the C-SAH. The average daily exergy efficiency, presented in Fig. 14b, additionally demonstrates the benefit of the M-SAH. Efficiency improves by 45.5% under natural convection, going from 0.19% for the C-SAH to 0.28% for the M-SAH. Efficiency increases consistently for all flow rates under forced convection, rising to 1.45% at 0.0046 kg/s compared to 0.87%, 1.82% at 0.008 kg/s compared to 0.94%, and 2.13% at 0.012 kg/s compared to 1.03%. These findings show that, in every scenario studied, the recycled fin design successfully improves hourly and daily exergy performance. The absolute values of the exergy efficiency observed in this study, (peaking at 2.80%, exhibit relatively low; however, this is an inherent thermodynamic characteristic of solar air heating systems instead of a design flaw. The main reason is the high-grade nature of the solar input. Based on the Petela model (Eq. 9), treating the sun as a black body at 5777 K yields a Carnot efficiency of approximately 0.93, which means over 93% of the incident solar radiation is high-quality exergy, this leads to a massive exergy input denominator. On the other hand, the low heat capacity fluid of air decreases the physical exergy gained by the air despite significant increases in temperature. Additionally, the heat transfer through the large temperature gradient between air and the absorber plate, in addition to frictional pressure drops introduced by the fins, results in substantial exergy destruction.

### Economic, energy and environmental results

**Fig. 15:**
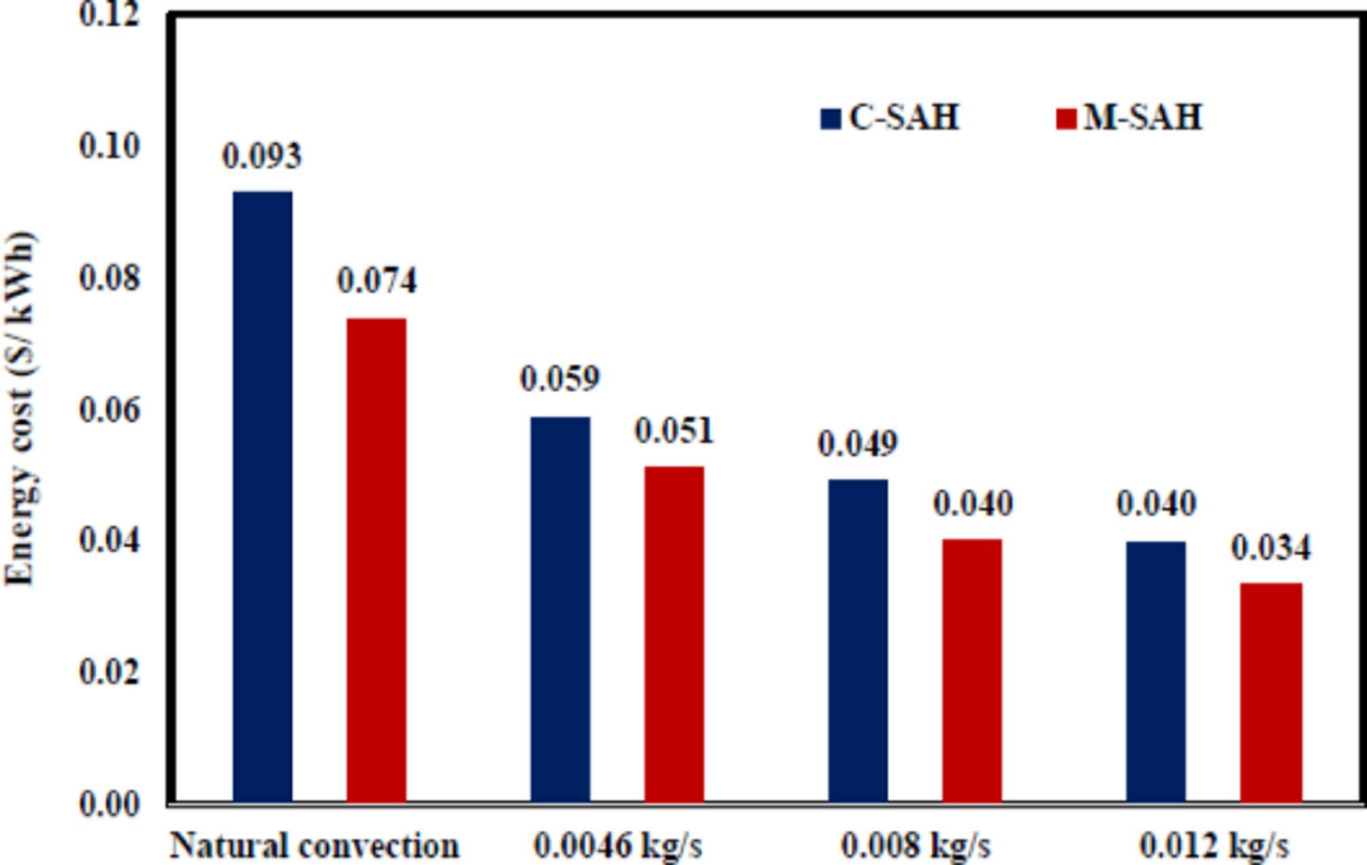
Energy cost of conventional and modified SAHs under natural and forced convection operating conditions.

The results of the economic parameter in terms of energy cost for each C-SAH and M-SAH are presented in Fig. 15. The results show that M-SAH achieves a lower energy cost than C-SAH under all operating conditions, in agreement with its superior thermal performance discussed earlier. Under natural convection, the energy cost decreases from 0.093 $/kWh for the C-SAH to 0.074 $/kWh for the M-SAH, indicating a cost reduction of 20.69%. When forced convection is applied, the energy cost is reduced from 0.059 to 0.051 $/kWh at 0.0046 kg/s, from 0.049 to 0.040 $/kWh at 0.008 kg/s, and from 0.040 to 0.034 $/kWh at 0.012 kg/s, corresponding to reductions of 13.15%, 18.73%, and 15.94%, respectively. These results confirm that integrating recycled aluminum fins enhances the economic energy costs of the solar air heater by lowering the unit energy cost under all tested flow conditions.

Table 5 presents the embodied energy, energy payback time, and exergo-economic performance of the conventional solar air heater (C-SAH) and the modified solar air heater (M-SAH) under various mass flow rates. The M-SAH offers much more annual usable energy and exergy in all scenarios studied, while having a somewhat higher capital cost and embodied energy because of the recycled aluminum fins. Across all studied mass flow rates, these enhancements result in noticeably reduced energy and exergy payback times for the M-SAH. The EPBTen drops from 31.4 years for the C-SAH to 15.4 years for the M-SAH under natural convection, the EPBTex drops from 2.4 to 2.0 years, and Rex marginally rises from 0.2 to 0.2 kWh/$. With forced convection, increasing the air mass flow rate further enhances performance. At 0.0046 kg/s, EPBTen reduces from 8.2 to 20.0 years and EPBTex from 1.1 to 0.9 years, with Rex rising from 0.6 to 1.1 kWh/$. At 0.008 kg/s, EPBTen decreases from 8.8 to 20.5 years and EPBTex from 0.9 to 0.7 years, while Rex improves from 0.6 to 1.2 kWh/$. At the highest mass flow rate of 0.012 kg/s, EPBTen falls from 9.6 to 29.9 years and EPBTex from 0.7 to 0.6 years, with Rex reaching 1.7 kWh/$ compared with 0.7 kWh/$ for C-SAH.

**Table 5:** Embodied energy, energy payback time, and exergoeconomic for all SAHs.

Parameter		CAT ($)	Embodied energy (kWh)	Annua useful energy (kWh/year)	Annual useful exergy (kWh/year)	EPBTen year	EPBTex year	Rex (kWh/$)
Natural convection	C-SAH	83.0	258.1	104.3	1.7	2.4	142.7	0.2
	M-SAH	89.0	307.5	146.1	2.5	2.0	117.6	0.2
0.0046 kg/s	C-SAH	83.0	258.1	238.3	8.2	1.1	31.4	0.6
	M-SAH	104.0	307.5	348.2	20.0	0.9	15.4	1.1
0.008 kg/s	C-SAH	83.0	258.1	284.3	8.8	0.9	29.2	0.6
	M-SAH	104.0	307.5	444.0	20.5	0.7	15.0	1.2
0.012 kg/s	C-SAH	83.0	258.1	350.5	9.6	0.7	26.8	0.7
	M-SAH	104.0	307.5	529.1	29.9	0.6	10.3	1.7

**Fig. 16:**
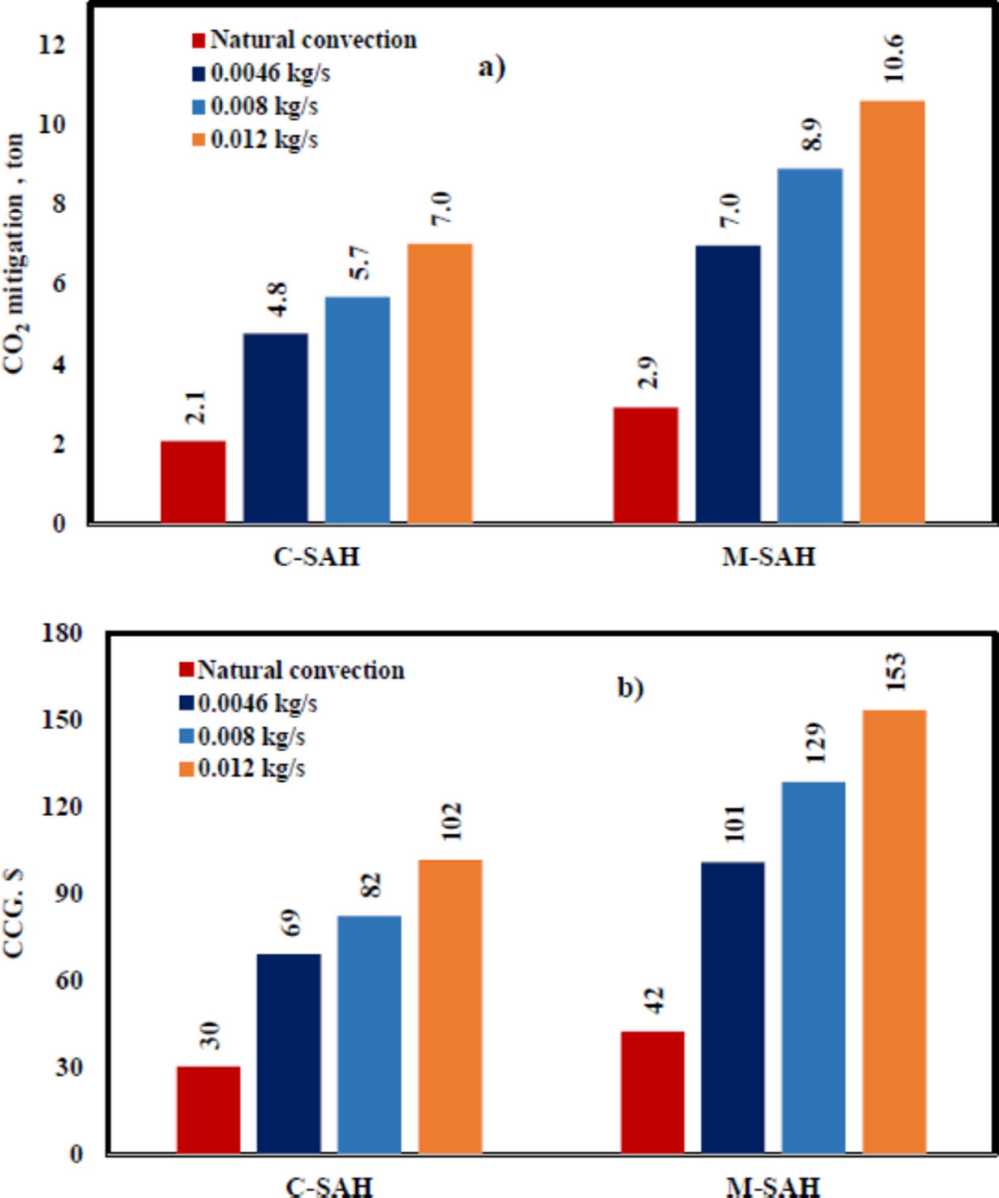
Enviro-economic assessment for the SAHs under different mass flow rates (**a**) CO₂ mitigation and (**b**) Carbon credited gain (CCG, $) for the C-SAH and M-SAH.

Figure 16a illustrates the CO₂ mitigation potential of the C-SAH and M-SAH under different mass flow rates. Under natural convection, the M-SAH reduces 2.9 tons of CO₂ compared with 2.1 tons for the C-SAH. The environmental advantage of the modified design becomes more pronounced at higher mass flow rates. Where, at 0.0046 kg/s, the M-SAH mitigates 7.0 tons of CO₂ versus 4.8 tons for the C-SAH, increasing to 8.9 and 10.6 tons at 0.008 kg/s and 0.012 kg/s, respectively, compared with 5.7 and 7.0 tons for the conventional C-SAH. Furthermore, the M-SAH consistently provides higher economic benefits through carbon credits (see Fig. 16b). Where, at natural convection, the CCG rises from $30 for the C-SAH to $42 for the M-SAH. At 0.0046 kg/s, the M-SAH reaches $101 compared with $69 for the C-SAH, and at 0.008 kg/s and 0.012 kg/s, the M-SAH achieves $129 and $153, respectively, while the C-SAH attains $82 and $102. Which confirms that, the M-SAH yields substantial economic and environmental benefits attributable to increased carbon credit potential.

### Sustainability indexes

**Fig. 17:**
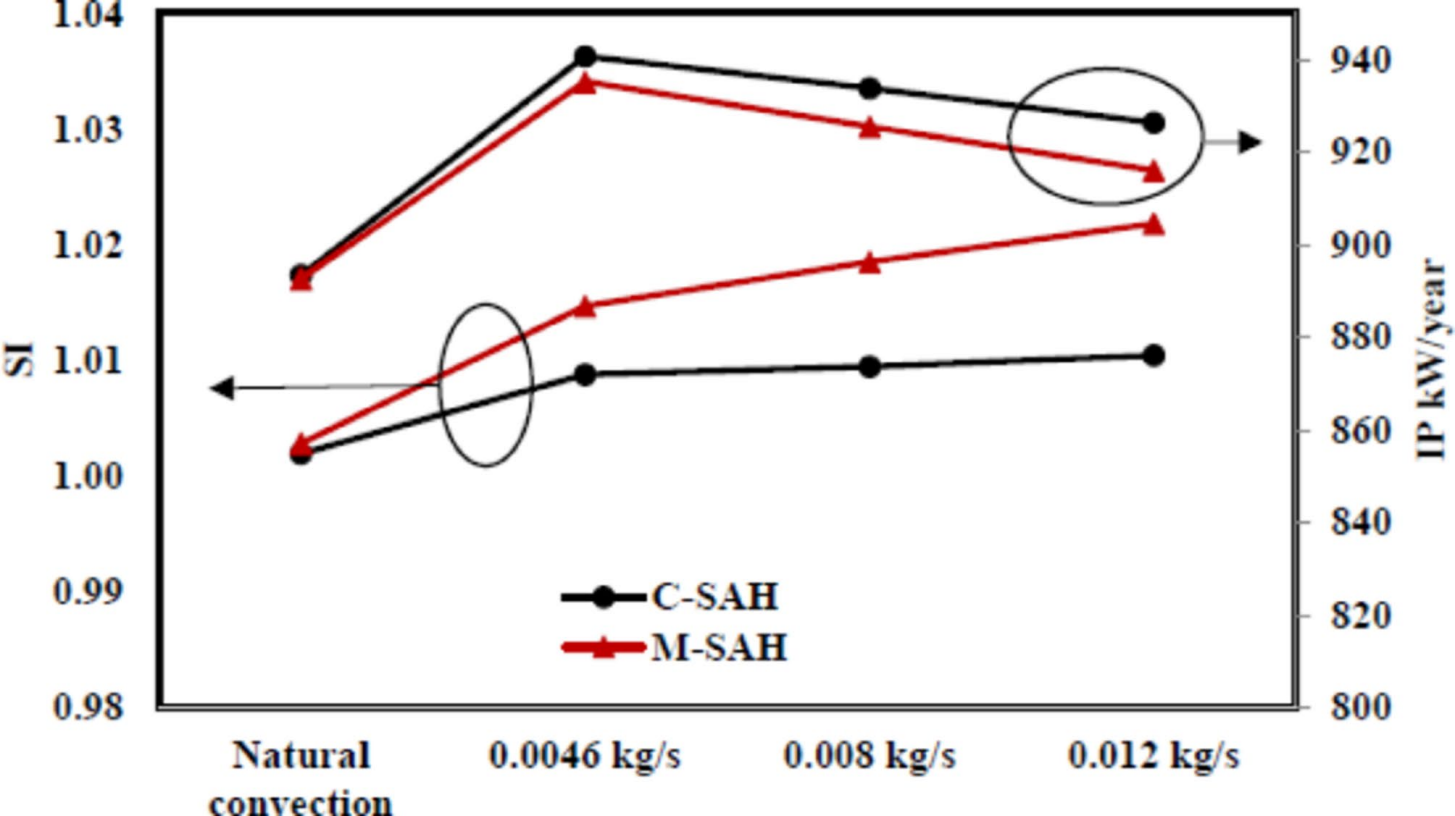
Variation of SAH sustainability index (SI) and improvement potential (IP) for C-SAH and M-SAH under natural and forced convection operating conditions.

The calculated sustainability index (SI) and improvement potential (IP) for both C-SAH and M-SAH under natural and forced convection conditions are illustrated in Fig. 17. In natural convection, both SAHs exhibit an SI of 1.00 and an IP of 893 kW/year, indicating similar baseline sustainability. The recycled aluminum fins in the M-SAH slightly improve the SI under forced convection, reflecting enhanced heat transfer and more effective energy utilization. At a mass flow rate of 0.0046 kg/s, the SI increases to 1.01 for both SAHs, while the IP reaches 941 kW/year for the C-SAH and 935 kW/year for the M-SAH. At 0.008 kg/s, the M-SAH achieves an SI of 1.02 and an IP of 925 kW/year, slightly higher than the C-SAH. At the highest flow rate of 0.012 kg/s, the M-SAH maintains a higher SI of 1.02, while the IP slightly decreases to 916 kW/year compared with the C-SAH, which has an SI of 1.01 and an IP of 926 kW/year.

### Results of AI model

**Fig. 18:**
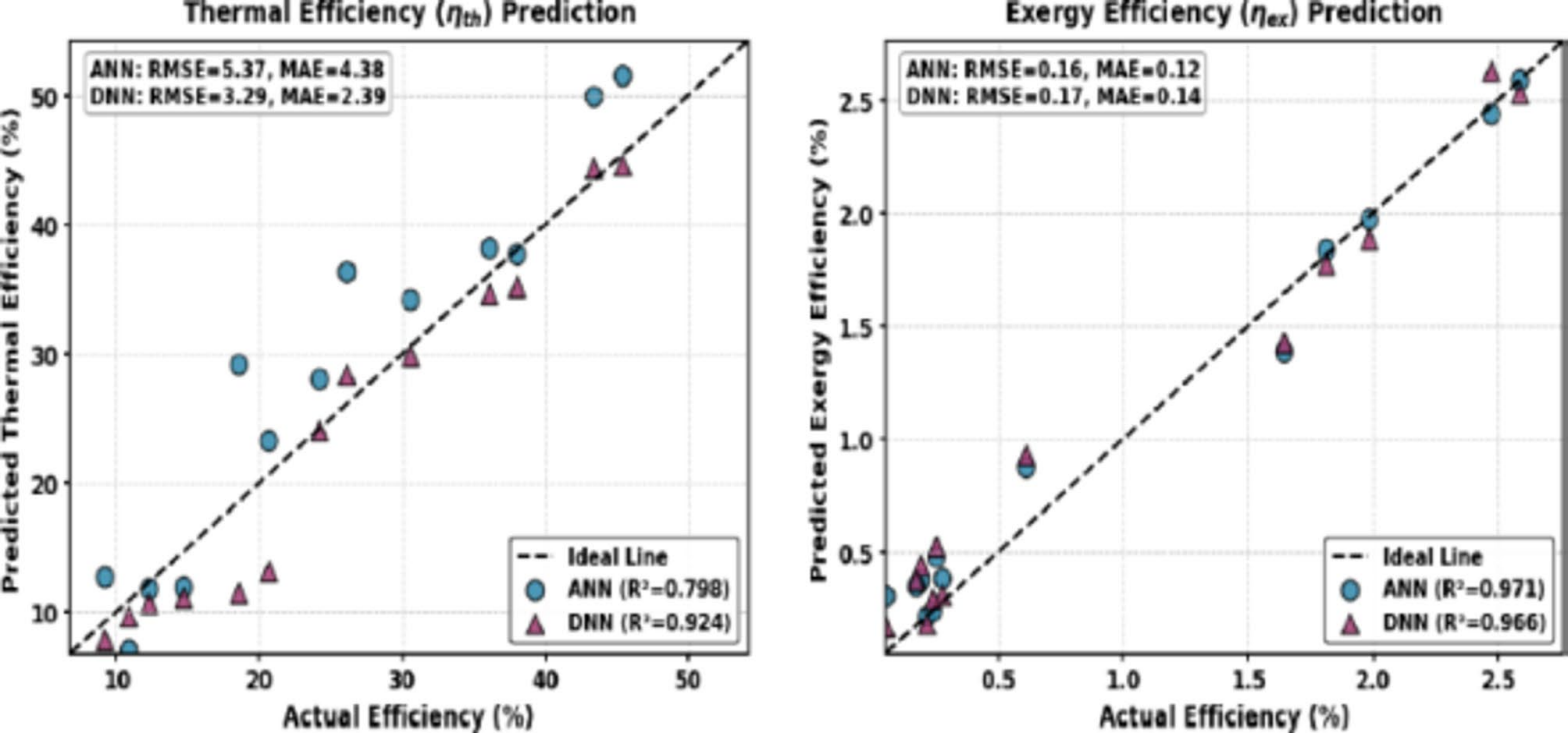
Scatter plots for thermal and exergy efficiencies.

Figure 18 presents the scatter plot analysis that compares the experimental values with AI-predicted values of thermal and exergy efficiencies. The figure reflects high performance for both AI models in predicting each thermal and exergy efficiency for SAHs, with clear differences indicating the nature of each efficiency output. Regarding thermal energy efficiency, the DNN model significantly outperformed the ANN, exhibiting a higher R² of 0.9243 vs. 0.7978, RMSE of 3.29% vs. 5.37%, and MAE of 2.39% vs. 4.38%. This elucidates the ability of the additional layers in DNN to capture the complex nonlinear dynamics of heat transfer in SAH systems. For the exergy efficiency (ηex), both models achieved high performance with R² > 0.96, with a slight advantage for the ANN R² of 0.9710 vs. 0.9658 for the DNN. This is due to the fact that exergy efficiency has a more linear relationship with input factors, particularly solar radiation intensity and ambient temperature, which makes the simpler ANN model sufficient without the added complexity of the DNN. The observed difference in performance illustrates that the selection of network architecture should rely on the nature of the physical phenomenon under study. The DNN is more suitable for systems that involve complex nonlinear dynamics, such as forced-flow heat transfer, benefiting from the added depth, while simpler models such as ANN can be appropriate for systems involving quasi-linear phenomena, such as exergy generation, to avoid overfitting. These results demonstrate that both models provide reliable ways to predict the performance of SAHs, with significant guidance for the optimal choice of the model based on the specific engineering objective. The statistical reliability of the AI approach was further validated by determining the 95% confidence intervals (CIs) based on the residual errors from the 5-fold cross-validation. For thermal efficiency predicted by the DNN model, the 95% CI margin of error was approximately ± 6.45%, derived from the RMSE of 3.29%. While exergy efficiency predicted by the ANN model achieved a highly narrow 95% CI of ± 0.30%, based on a residual standard deviation of 0.155. These tight confidence bounds demonstrate that the vast majority of the experimental data points fall well within the acceptable statistical uncertainty limits, which supports the generalization ability and robustness of the proposed models. To reveal the superiority of the DNN compared to ANN for thermal efficiency, it is important to consider the structural differences based on heat transfer physics. The thermal efficiency of SAH is governed by highly nonlinear, multivariable interactions, mainly the complex relationship between absorber temperature, mass flow rate, and the turbulence created by the interlocking fins. The structure of the ANN model has a limited factor capacity of around 800 trainable parameters, which is enough for quasi-linear relationships like exergy efficiency but limits its ability to capture these high-order thermal interactions. On the other hand, the architecture of the DNN increases the factor capacity to around 3,100. This additional depth enables hierarchical feature extraction, which allows the network to successfully model the complex, secondary flow dynamics, and non-linear boundary layer disruptions. Coupled with a higher dropout rate of 0.35 to avoid overfitting on the limited dataset, this deeper architecture was uniquely able to capture the intricate thermodynamic behavior of the modified SAH.

**Fig. 19:**
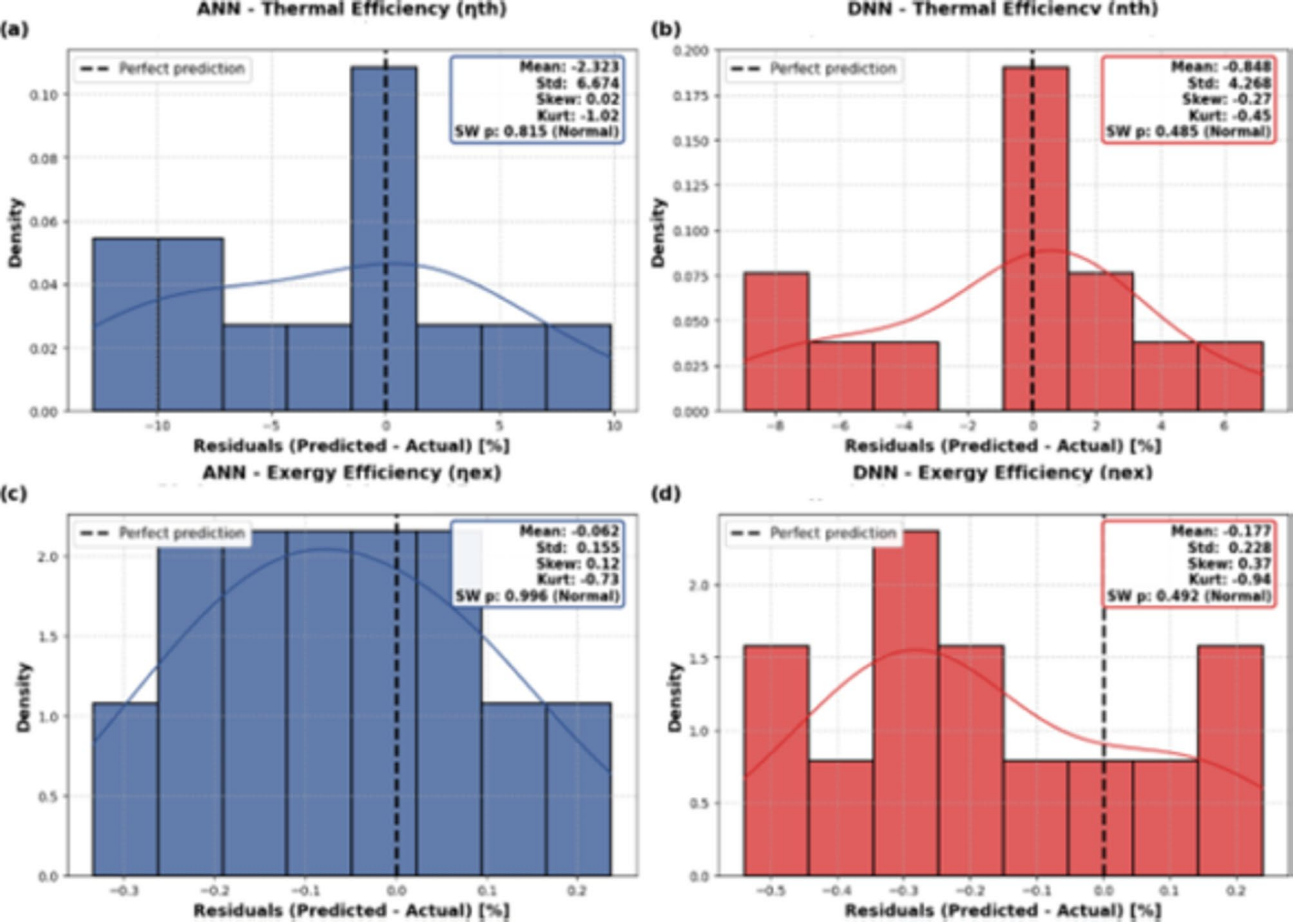
The residual histograms for ANN and DNN models.

The results of the residual analysis indicated in Fig. 19 show the prediction errors of two models for the thermal and exergy efficiencies of SAH, with comprehensive statistical indicators elucidating the nature of the error distribution and the model stability. The prediction of thermal efficiency using the ANN model (plot a), the mean residue (Mean) of − 2.323 illustrates an obvious negative bias, which means that the model tends to underestimate the actual values. The relatively high standard deviation (Std) of 6.674 reveals clear error dispersion and prediction instability across different operating conditions. The skewness value (Skewness) of 0.02, near zero, shows a near-symmetric distribution, while the kurtosis value (Kurtosis) of − 1.02 indicates a relatively flat distribution without sharp peaks, meaning the absence of extreme errors. The Shapiro-Wilk p-value (SW) of 0.815 is higher than 0.05, which illustrates that the distribution exhibits a statistically normal pattern despite the wide dispersion. For thermal efficiency predicted by the DNN model (plot b), the mean residual value reduced to − 0.848, illustrating a clear decrease in bias and increased prediction accuracy.

The std also reduced to 4.268, which shows enhanced stability and decreased error dispersion. The skewness value of −0.27 highlights a slight tendency towards negative values, while the kurtosis of −0.45 illustrates a near-normal distribution without sharp tails. The SW value of 0.485 also reflects that the distribution is statistically normal. Regarding exergy efficiency using the ANN (plot c), the mean residual value was −0.062, which is very near to zero, illustrating almost no bias. The very low std of 0.155 indicates high accuracy and obvious stability of prediction. The skewness value of 0.12 illustrates a nearly symmetrical distribution, and the kurtosis of −0.73 shows a moderate distribution without extreme values. The very high SW value of 0.996 strongly demonstrates that the errors exhibit a normal distribution. In the case of the DNN exergy efficiency (plot d), the mean residual was −0.177, which is close to zero with a low std of 0.228, this reflects good prediction accuracy, despite being slightly higher than that of the ANN. The skewness of 0.37 shows a slight bias towards positive values, while the kurtosis of −0.94 indicates a relatively flat distribution. The SW value of 0.492 remains above 0.05, which emphasizes the normal distribution of errors. The results of this analysis emphasize that the DNN model is more stable in predicting thermal efficiency because of its ability to capture nonlinear complexities, while the ANN model had the best stability and accuracy in exergy efficiency owing to the simplicity of the input-output relationship in this context.

**Fig. 20:**
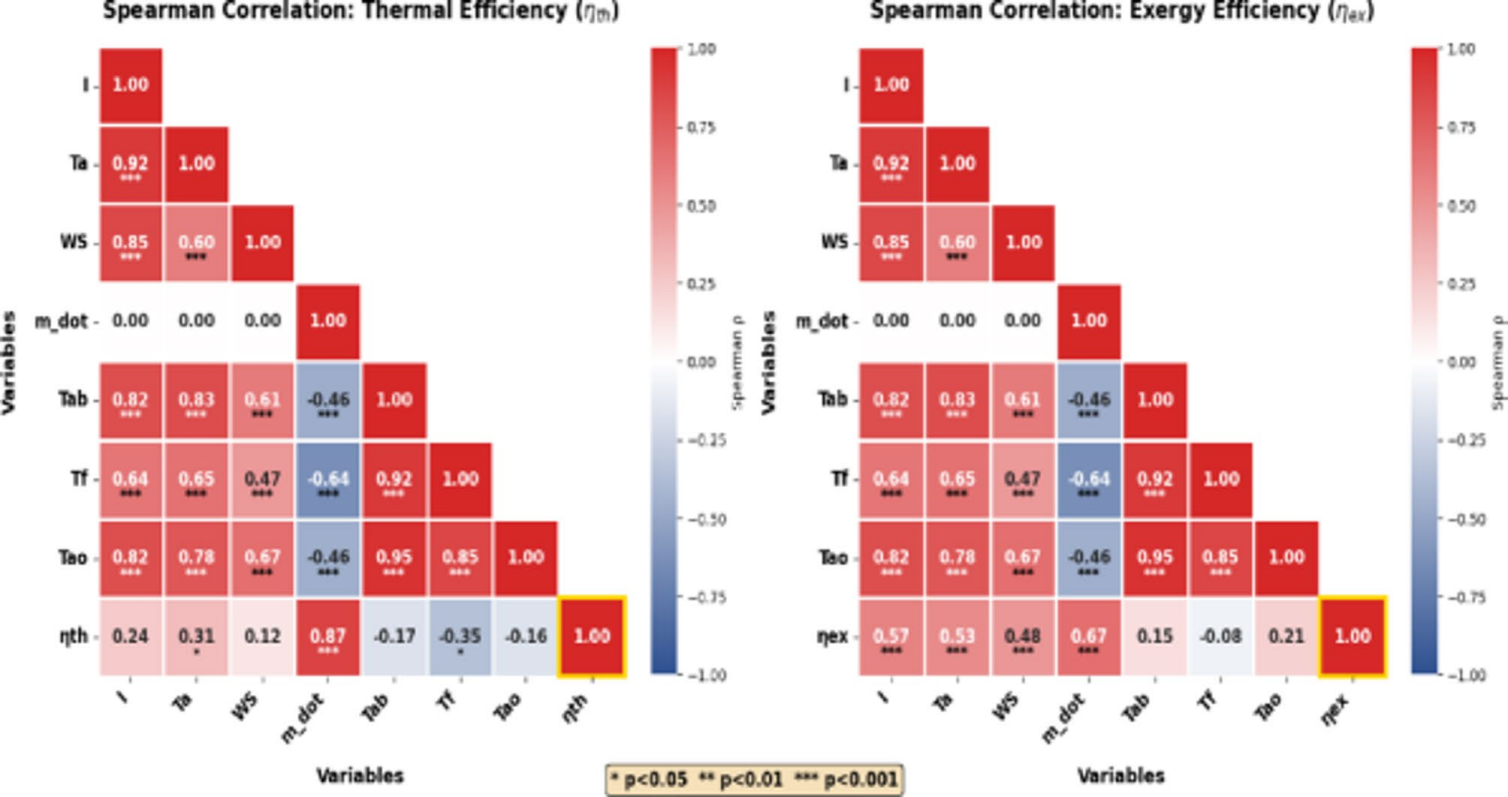
Spearman heatmaps for thermal and exergy efficiencies.

Figure 20 presents the Spearman heat maps for thermal and exergy efficiency. The Spearman correlation significantly illustrates the direction and strength of the relationship between operational factors and each energy and exergy efficiency. The color values indicate the varying sensitivity of each efficiency to the operating parameters. These heat maps elucidate the fundamental differences between the quantity and quality of energy in SAH. For thermal energy efficiency, mass flow rate showed the strongest effective parameter with a high positive correlation (ρ) of 0.871 and *p* < 0.001, demonstrating that the increase in air flow rate directly enhances forced convection heat transfer and decreases heat loss, thereby improving the quantitative efficiency of the SAH. On the contrary, the temperatures of hot surfaces, mainly absorber plates and fins, elucidate weaker or relatively negative correlations (with ρ of −0.46 and − 0.64, respectively) with thermal efficiency. It is essential to illustrate that this negative correlation is primarily an evidence of increased thermal losses to the surrounding, rather than a limitation in the design capability of the fins to promote heat transfer. Physically, with the increase in the absorber and fin temperatures, typically occurring at higher solar irradiance or lower mass flow rates, the temperature gradient between ambient air and these hot surfaces significantly increases. This leads to higher rates of convective and radiative heat losses from the collector cover to the surroundings. Therefore, a larger portion of the absorbed solar energy is wasted to the environment instead of being transferred to the air, inherently decreasing the overall thermal efficiency. This thermodynamic reality is further supported by the fact that the M-SAH operates at lower fin and absorber temperatures than the C-SAH under identical conditions, which demonstrates that the proposed fins effectively extract heat, but the main penalty of high-temperature top losses still affects the overall efficiency trend. Ambient air temperature and solar radiation indicate moderate positive impacts, which demonstrate that enhanced environmental conditions contribute to improved thermal efficiency, but to a lesser extent than the effect of mass flow. On the other hand, the exergy efficiency (η_ex_) highlights that the effect of physical variables was quite different: solar irradiance (I) and ambient temperature emerged as moderately influential variables with ρ of 0.575 and 0.533, respectively, and *p* < 0.001, confirming that the quality of energy is directly influenced by the quality of the heat source according to Carnot’s law (1 - T₀/T). Wind speed (WS) had a moderately positive impact with ρ of 0.483 and *p* < 0.001. This surprising impact may be owing to its role in enhancing heat transfer conditions and decreasing surface temperatures, thus preserving the quality of the energy produced. The most significant difference was that the exergy efficiency was less affected by fin temperature and absorber plate, with weak or insignificant correlations, which explains that the thermal design of the fins improves qualitative efficiency less than it influences quantitative efficiency. The results obtained reveal that enhancing the performance of SAH requires a careful understanding of the differences between the two efficiencies. As the increase in mass flow enhances both efficiencies, controlling ambient temperature and wind is more important for enhancing energy quality than quantity (heat).

**Fig. 21:**
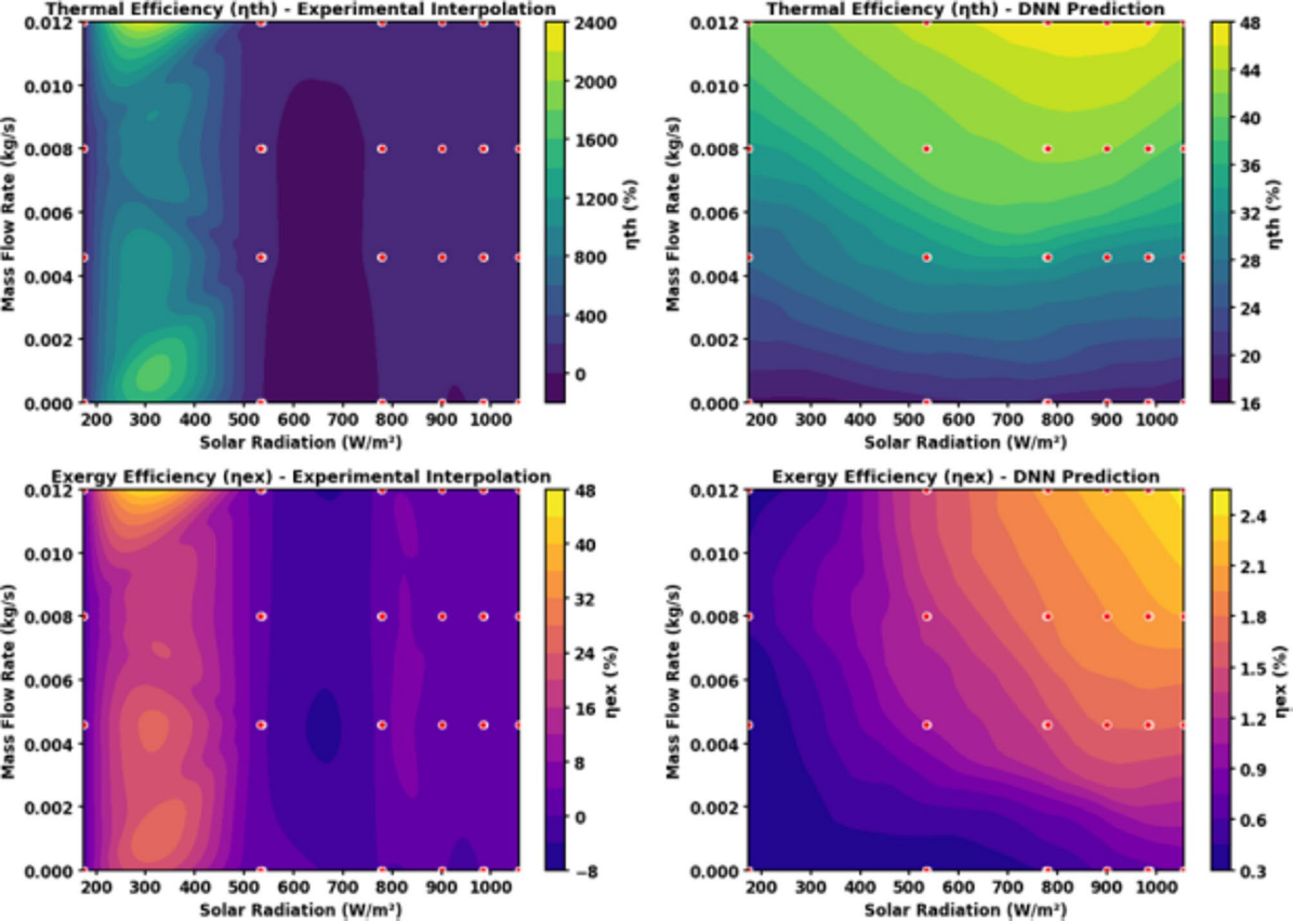
Contour maps for thermal and exergy efficiencies.

The contour maps indicated in Fig. 21, as shown by the 2-D response surface between solar irradiance and each air mass flow rate, thermal, and exergy efficiencies. These contours elucidate critical operating patterns that can help in the design and optimization of SAHs. The results of these contours were derived via two complementary techniques: (1) direct interpolation of experimental results using cubic interpolation to accurately represent the actual measurements, and (2) the employment of a DNN to predict efficiency values on a fine mesh (80 × 80 points) with auxiliary data. The contours show an important positive correlation between airflow and irradiation and thermal efficiency (ηth). Only at high irradiance (more than 900 W/m²) and high forced flow (0.012 kg/s) can an efficiency of more than 45% be reached. Even at maximum solar irradiation, the natural convection SAH, which has a flow rate of zero, exhibits a maximum of no more than 21%, underscoring the critical role that forced flow plays in competitive performance.

A delicate thermal balance between enhanced heat transfer and increased frictional losses is indicated by the fact that energy efficiency (ηex) is more sensitive to air flow rate than thermal efficiency. It peaks at about 2.8% at high solar irradiance and medium-to-high flow, while yields above 0.008 kg/s are significantly reduced at moderate solar irradiance. With a solar radiation at 800–1000 W/m² and a flow rate of 0.008–0.010 kg/s, the contour maps identified the ideal operating region for maximizing performance. A critical practical recommendation was made below 600 W/m², the benefit of forced convection is insignificant when compared to fan consumption, while above 1000 W/m², higher flow rates are necessary to prevent thermal saturation.

**Fig. 22:**
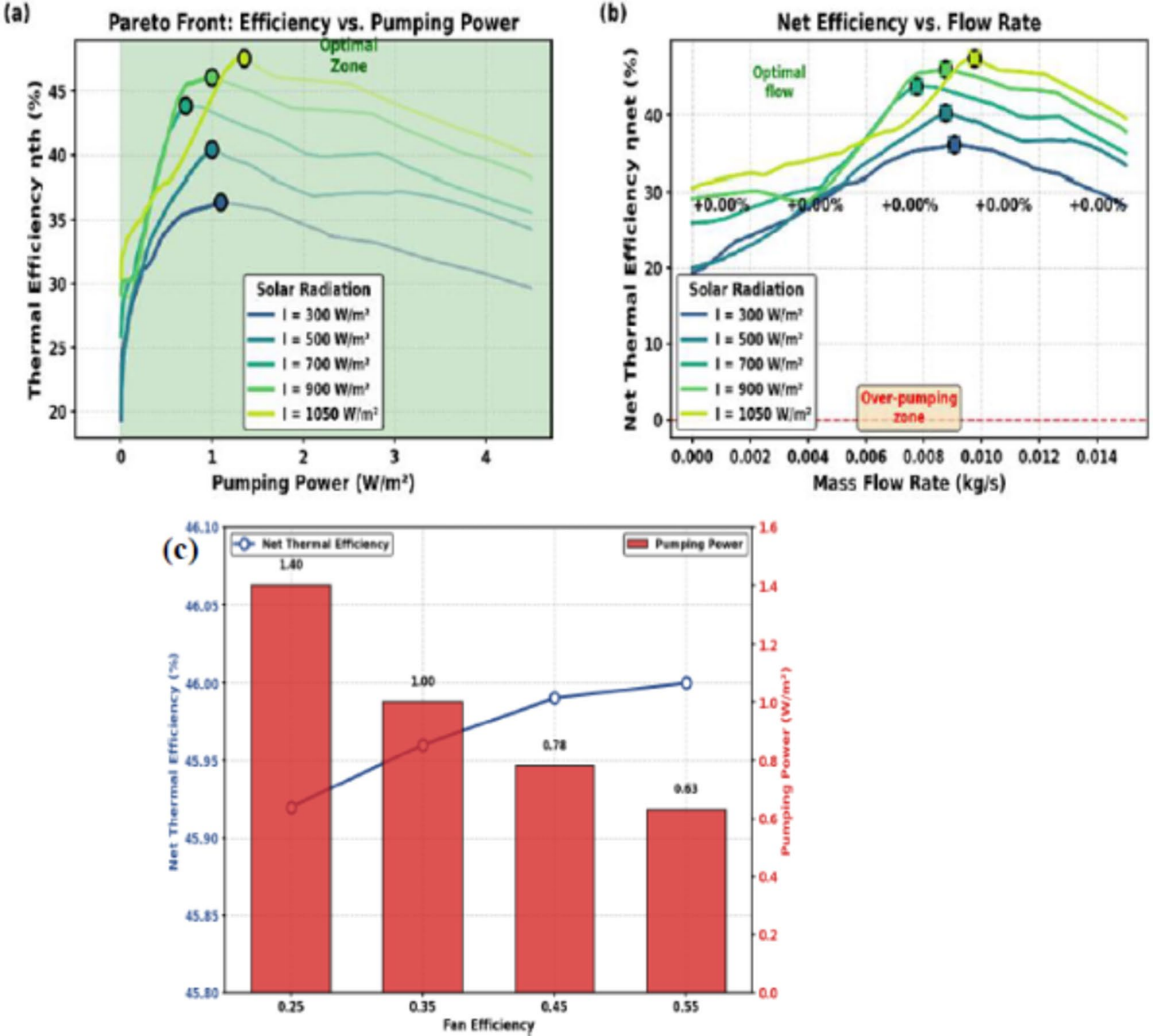
Perto optimization of SAH operation.

The outcomes of the multi-objective Pareto optimization study are displayed in Fig. 22. Important engineering insights that modify SAH operational settings have been revealed by this approach. The figure demonstrates that optimizing apparent thermal efficiency by itself leads to erroneous operational choices. This is because operating at the maximum thermal efficiency point results in an 8–15% loss of net potential energy due to the fan’s excessive power consumption, a factor that traditional efficiency analysis ignores. A critical threshold of 250 W/m² separates the two operating zones: forced flow (optimal above it) and natural load (optimal below it). The analysis identifies the optimal Pareto region that balances thermal gains and pumping costs, showing that the optimal air mass flow follows a non-linear relationship with solar irradiance (ṁ_opt_ ≈ 0.012 kg/s at high irradiance). Because fan consumption exceeds heat gain, the Pareto chart (figure A) shows a sharp decline in efficiency above 0.010 kg/s, with each subsequent 0.001 kg/s increase resulting in net energy losses. The net efficiency versus flow rate (plot B) elucidates a golden operating region, with mass flow rates of 0.008–0.010 kg/s at ² solar radiation of 800–1000 W/m, achieving maximum net energy. Plot c indicates the relationship between fan efficiency and its effect on the net thermal efficiency of SAH, confirming a delicate balance between decreasing energy consumption and enhancing fan performance. A clear enhancement was observed in pumping power from 1.40 W/m² to 0.63 W/m² with an increase in fan efficiency from 0.25 to 0.55, along with a slight reduction in net thermal efficiency from 46.06% to 45.93%. These results elucidate that the increase in fan efficiency results in a disproportionate decrease in energy losses due to air pumping, as energy consumption reduced by approximately 55% while net efficiency was only minimally influenced by 0.13%. The relationship between energy consumption and fan efficiency, indicated by the blue line, is inversely proportional, while the red bars highlight that net efficiency slightly reduces as fan efficiency enhances, which suggests an optimal balance at a fan efficiency of 0.45, where net efficiency reaches 45.96%, with an energy consumption of 0.78 W/m². These findings elucidate that selecting a high-efficiency fan, greater than 0.45, achieves an optimal balance between decreasing energy consumption and enhancing the overall performance of SAH, consequently decreasing long-term operating costs without clearly affecting net efficiency. It is essential to indicate that the values of aerodynamic efficiency of the fan, ranging from 0.25 to 0.55 that were used in the Pareto optimization were not directly measured. They were assumed based on the technical specifications of the manufacturer and data of typical performance for small-scale 12 V DC axial fans. This range was used as a parametric factor to theoretically estimate the sensitivity of the net energy balance to variations in fan performance, while the consumption of baseline auxiliary electrical power was evaluated based on the operational characteristics of the fan to ensure the accuracy of the net efficiency calculations.

### Comparative study

Table 6 provides a comparison between the obtained results and those of previous studies, focusing on different shapes of fins. The table reveals the distinct novelty of this study represented by the use of the 3D interlocking U-shaped profiles. Unlike other geometries, such as rectangular and V-corrugated, which only provide external surfaces, the ICF simultaneously provides external surfaces and produces confined internal channel walls with integrated ribs. This unique geometry maximizes the convective heat transfer area (ratio of 1.5), disrupts the boundary layer, induces strong secondary flow structures, and increases turbulence intensity within the air stream, which results in a peak thermal efficiency of 48.6% and exergy efficiency of 2.80% without the requirement for complex multi-pass setups or additional PCM. Regarding the increase in surface area, the proposed ICF achieves a surface area enhancement ratio of 1.5 (increasing the effective heat transfer area from 0.60 m² to 0.92 m²) compared to 1.19 for Standard Rectangular^52^ and 1.4–1.5 for V-Corrugated Fins^53^. Furthermore, the ICF is characterized by its 3D modular interlocking U-shaped design rather than purely 2D extensions of rectangular or other shapes.

**Table 6:** Comparison with previous studies.

SAH type	Fins specifications	Key results	Refs.
Single pass	-Rectangular fins filled with PCM -The height was 0.06 m and 0.09 m and width 0.015 m and 0.025 m	- The maximum energy and exergy efficiency of a finned PCM was 69.7% and 13.6%., respectively.	^52^
Single pass	- V-corrugated fins with a height of 20 mm and a pitch of 50 mm	- The efficiency is improved by 25–30.5%	^53^
Semi-cylindrical	- Tubular, radial tubular, constructed tubular with 2 mm thickness and 20 mm height	- The thermal efficiency increased by 13.5% using constructed tubular	^67^
Single pass	-Double triangular fins with dimensions of 77 mm × 40 mm × 18 mm	- The maximum thermal efficiency was 86.4%	^68^
Double pass	-Airfoil fins with length of 17.68 mm	- The maximum improvement in the relative Nu was 4.23 and 2.51 times	^69^
Double pass	− 40 staggered-diamond shaped fins with height, width, and length of 30 mm, 50 mm, and 50 mm	- The highest thermal efficiency was 59.34%	^70^
Single pass	- Recycled Interlocking Channel Fins - U-shape with length of 4 cm, a width 4 cm, and a height of 4 cm	- The highest thermal and exergy efficiency were 48.6%and 2.80%, respectively.	**Current study**

## Conclusions

- The air heating performance of a conventional solar air heater (C-SAH) and a modified solar air heater (M-SAH) with fins made from recycled aluminum waste obtained from used window and door frames was experimentally evaluated in this study. At air mass flow rates of 0.0046, 0.008, and 0.012 kg/s, under the same climate conditions, the performance under natural convection was also compared with forced convection operation. The enhanced heat transfer area and better air–absorber interaction are the primary reasons why the upgraded solar air heater outperforms the standard configuration in terms of thermal performance, according to the comparative results. Furthermore, compared to natural convection, where the airflow rate greatly influences the thermal behavior, forced convection operation has unique performance characteristics. The following is a summary of the study’s main findings:
- The M-SAH achieves its highest outlet air temperature under natural convection at 84 °C, while under forced convection, the peak outlet temperature decreases with increasing mass flow rates of 77, 67, and 60 °C at 0.0046, 0.008, and 0.012 kg/s, respectively. • The reduction in absorber plate temperature observed in the M-SAH relative to the C-SAH ranges between 5% and 10%. • The M-SAH with recycled aluminum fins achieves average energy and exergy efficiencies of 48.36% and 2.13% at 0.012 kg/s, compared with 36.7% and 1.03% for the C-SAH. Increasing the mass flow rate from 0.0046 to 0.012 kg/s improves energy efficiency from 28.78% to 48.36% and exergy efficiency from 1.45% to 2.13% for the M-SAH.

• The M-SAH achieves a lower energy cost than the C-SAH, decreasing from 0.093 to 0.074 $/kWh under natural convection and to 0.051, 0.040, and 0.034 $/kWh at mass flow rates of 0.0046, 0.008, and 0.012 kg/s, respectively. • The M-SAH outperforms the C-SAH in CO₂ mitigation and carbon credits, increasing from 2.9 vs. 2.1 tons and $42 vs. $30 under natural convection to 7.0, 8.9, and 10.6 tons and $101, $129, and $153 at 0.0046, 0.008, and 0.012 kg/s, respectively. • The AI models exhibited high prediction accuracy; for thermal efficiency, the DNN model achieved the highest accuracy with an R² of 0.924, while the ANN model performed best in predicting exergy efficiency with an R² of 0.971.

### Limitations and future work

Despite the promising outcomes of this study, it exhibited some limitations that should be addressed in future work. The experiments were performed over four specified days under clear skies; this short-term investigation does not identify the transient thermal response of the system under fluctuating weather. This requires conducting long-term outdoor investigations under different ambient conditions to evaluate the system’s thermal inertia and assess the long-term durability of aluminum fins. The study doesn’t include direct measurements of pressure drop (ΔP) and traditional thermo-hydraulic indicators, such as the friction factor and PEC. Therefore, future studies should consider pressure drop measurements to offer a comprehensive fluid dynamic characterization of the interlocking fins. Despite this study identifying optimal operating conditions, these are specific to lab-scale systems. These absolute flow rates cannot be directly extrapolated to larger industrial systems. Therefore, future studies should identify these optimal conditions for larger-scale applications using dimensionless parameters, such as mass flux and specific pumping power, to accurately scale the findings. The use of outlet temperature as an input variable may lead to information leakage and an overestimation of the predictive ability of the AI models. This requires future AI-based optimization to use only input parameters, such as ambient conditions, geometric characteristics, and mass flow rate, to avoid any possibility of information leakage and to assess the generalization ability of the models under unseen operating conditions. The Computational Fluid Dynamics (CFD) simulations are also recommended to explicitly indicate the flow structures within the interlocking channels, mainly turbulence generation, boundary layer disruption, and secondary flow formation.

## Data Availability

The data that support the results of this work are not openly available due to institutional and intellectual-property restrictions associated with the research implementation and its future extension.
